# Dual inhibitors of *Pseudomonas aeruginosa* virulence factors LecA and LasB†

**DOI:** 10.1039/d4sc02703e

**Published:** 2024-07-22

**Authors:** Olga Metelkina, Jelena Konstantinović, Andreas Klein, Roya Shafiei, Mario Fares, Alaa Alhayek, Samir Yahiaoui, Walid A. M. Elgaher, Jörg Haupenthal, Alexander Titz, Anna K. H. Hirsch

**Affiliations:** a Helmholtz Institute for Pharmaceutical Research Saarland (HIPS), Helmholtz Centre for Infection Research (HZI) Campus E8.1 66123 Saarbrücken Germany alexander.titz@helmholtz-hips.de anna.hirsch@helmholtz-hips.de; b Deutsches Zentrum für Infektionsforschung (DZIF) Standort Hannover – Braunschweig, 38124 Braunschweig Germany; c Department of Chemistry, Saarland University 66123 Saarbrücken Germany; d Department of Pharmacy, Saarland University 66123 Saarbrücken Germany

## Abstract

Dual inhibitors of two key virulence factors of *Pseudomonas aeruginosa*, the lectin LecA and the protease LasB, open up an opportunity in the current antimicrobial-resistance crisis. A molecular hybridization approach enabled the discovery of potent, selective, and non-toxic thiol-based inhibitors, which simultaneously inhibit these two major extracellular virulence factors and therefore synergistically interfere with virulence. We further demonstrated that the dimerization of these monovalent dual inhibitors under physiological conditions affords divalent inhibitors of LecA with a 200-fold increase in binding affinity. The bifunctional LecA/LasB-blocker 12 showed superiority for the inhibition of virulence mediated by both targets over the individual inhibitors or combinations thereof *in vitro*. Our study sets the stage for a systematic exploration of dual inhibitors as pathoblockers for a more effective treatment of *P. aeruginosa* infections and the concept can certainly be extended to other targets and pathogens.

## Abstract 2

Dual inhibitors of two key virulence factors of *Pseudomonas aeruginosa*, the lectin LecA and the protease LasB, open up an opportunity in the current antimicrobial-resistance crisis.

## Graphical abstract

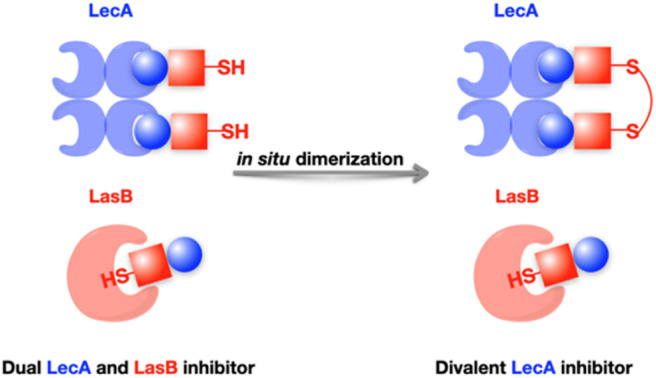

## Introduction

Bacterial resistance to antibiotics is a growing global health problem that urgently requires a solution.^1^ Disarming pathogens by targeting bacterial virulence factors has emerged as a new approach to fighting drug-resistant infections.^2–5^ Bacterial virulence factors such as toxins, adhesins, invasins, and quorum-sensing molecules play crucial roles in host colonization and infection promotion by suppressing the host immune defense.^6,7^ Reducing bacterial virulence *via* the inhibition of these factors also decreases the host's susceptibility to infection and allows the immune system to eliminate bacteria. Moreover, the inhibition of bacterial virulence factors mechanistically reduces the selection pressure for resistant mutants due to the ability of pathoblockers to disarm the pathogens without direct killing.^3,4^

*Pseudomonas aeruginosa* is a Gram-negative bacterium that is classified as critical on the WHO pathogen priority list and currently, numerous avenues of research are being explored in parallel to identify new therapeutics against this pathogen.^8,9^

Extracellular elastase LasB is a zinc-metalloprotease produced by *P. aeruginosa* and one of its pivotal virulence factors. Its importance for the overall pathogenicity of *P. aeruginosa* has been established and it is considered a valid drug target.^10^ The role of LasB in degrading the components of the connective tissue such as elastin and extracellular matrix components (ECMs, *e.g.*, collagen and laminin) facilitates host colonization.^11,12^ Besides this, LasB plays an important role in disrupting the host immune system through the degradation of immunoglobulins, cytokines and other immune factors.^13,14^ LasB is also known for hydrolyzing blood proteins, such as transferrin and lactoferrin, consequently leading to free-radical-induced cytotoxicity.^15,16^ In addition to the host substrates, LasB participates in the processing and activation of other bacterial components (LasA, leucine aminopeptidase, lysine endopeptidase and others), promoting the inflammation process.^17–19^

Another virulence factor, lectin LecA (PA-IL), is an extracellular galactophilic carbohydrate-binding protein expressed by *P. aeruginosa*, which mediates biofilm matrix formation and host colonization.^20,21^ It is responsible for bacterial adhesion interacting with the glycocalyx of mammalian cells.^22,23^ LecA also attenuates ciliary beating in human airways, preventing mucus clearance and inhibiting the growth of respiratory epithelial cells.^21,24,25^ On a molecular level, this lectin was shown to mediate bacterial uptake in a lipid zipper mechanism by binding to host glycolipids presented on its cell surfaces.^26^ Additionally, LecA enhances host cellular absorption of another virulence factor, exotoxin A, inducing a pathogenic effect on the intestinal epithelium and it increases the injury of the alveolar capillary barrier.^20,27^ It has been demonstrated that the inhibition of LecA reduces biofilm formation and the overall virulence of *P. aeruginosa*.^20^ Glycomimetics have the potential to act as promising pathoblockers.^28^ To increase the efficacy of LecA inhibition and overcome its moderate micromolar affinity to galactosides caused by a shallow binding pocket, multivalent ligands are often utilized.^29–33^

The crucial effects of these two proteins on the infection progress make them validated and attractive therapeutic targets. Simultaneously targeting both LasB and LecA is compelling for multiple reasons. The extracellular co-localization of LasB and LecA overcomes the major hurdle for many therapeutic molecules to penetrate the Gram-negative cell wall. Furthermore, the dual inhibitors can increase drug efficacy in comparison to combination therapy when the two separate therapeutic molecules are applied together.^34^ It is expected that a single drug acting on multiple targets possesses more predictable pharmacokinetic and safety profiles, lowers the probability of resistance development and avoids undesirable drug–drug interactions.^34^

In this work, we have chosen established inhibitors of LasB and LecA based on α-isobutyl/benzyl-*N*-aryl-mercaptoacetamide and phenyl β-d-galactoside, respectively, and merged them into one molecule that blocked LecA and LasB with moderate to high potency.^9,35–37,41^ In addition, by utilizing the inherently limited chemical stability of thiols and their tendency to form disulfides in an extracellular environment, highly potent divalent LecA-inhibitors were obtained. These disulfides are likely to be formed in the infection setting after saturating LasB *in situ*, yielding highly potent inhibitors of the second virulence factor LecA.

## Results and discussion

### Design of dual inhibitors

**Fig. 1:**
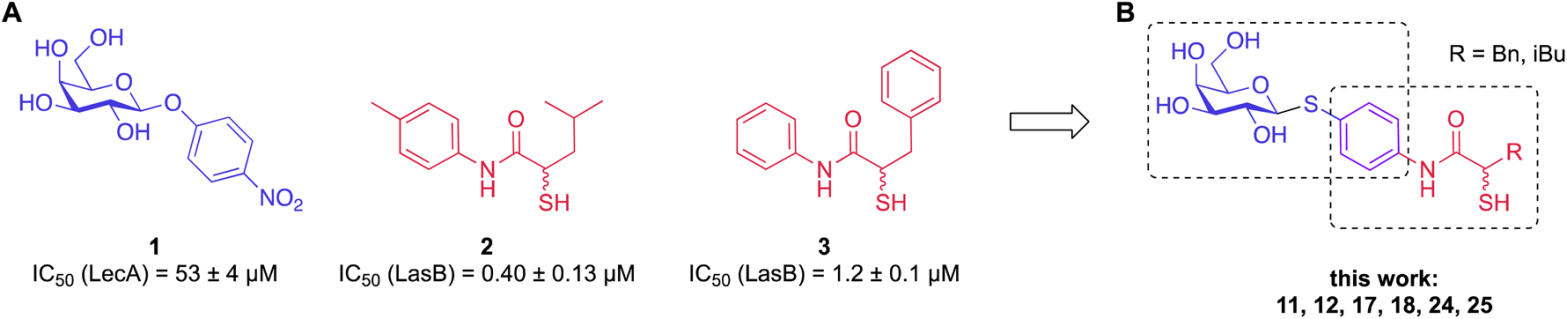
(A) Reported LecA and LasB inhibitors 1–3 and (B) their combination into dual inhibitors.

To develop dual inhibitors against *P. aeruginosa* virulence factors LasB and LecA, we selected previously developed thiol-based LasB ligands, which demonstrated high potency but also limited chemical stability due to their tendency to dimerize to disulfides under physiological conditions.^37^ It is known that the moderate micromolar potency of galactosides as LecA inhibitors can be significantly improved by utilizing divalent ligands, where two carbohydrate molecules are bridged with a linker to simultaneously bind to two sites in the LecA tetramer.^29,31,33,38^ Taking these facts into account, we combined nitrophenyl β-d-thiogalactoside 1 as a LecA inhibitor with the *N*-aryl-mercaptoacetamide-based LasB inhibitors 2 and 3 by merging their aryl moieties into dual inhibitors 11, 12, 17, 18, 24 and 25 (Fig. 1).^37,39–41^ The resulting compounds should be capable of efficiently inhibiting LasB in the initial thiol form, while the dimerization of excess ligand under physiological conditions at the infection site transforms them from moderately monovalent into more potent divalent LecA inhibitors.

**Fig. 2:**
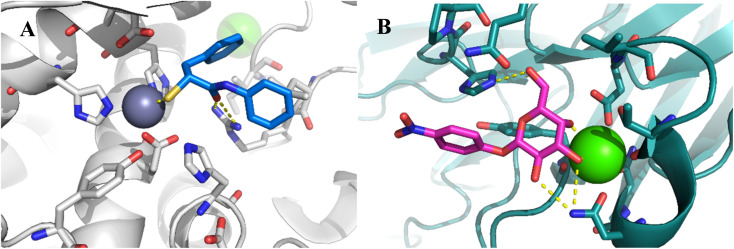
A) Crystal structure of LasB (gray) in complex with 3 (cyan) (PDB code: 7OC7);^37^ (B) crystal structure of LecA (petrol) in complex with 1 (magenta) (PDB code: 3ZYF).^45^

Our previously reported crystal structure of thiol 3 in complex with LasB suggested potential for further elongation of the molecule in the direction of the aniline ring (Fig. 2A).^37^ At the same time, the co-crystal structure of LecA with 1 showed the opportunity to modify the nitro group in the *para*-position of the phenyl ring without disturbing the crucial interaction of the sugar moiety in the binding pocket (Fig. 2B),^45^ which is backed by reported structure–activity relationships.^36,40,42,43^ In our hybridized molecules, the thiol group should maintain its crucial coordination to the zinc ion in the active site of LasB, while the galactoside will conserve the calcium(ii) chelation with its 3- and 4-hydroxy groups and the T-shaped CH–π interactions between the phenyl aglycon and His50 in the binding site of LecA.^40,44^

Further, we have shown that *N*-arylmercaptoacetamides have moderate redox stability and tend to oxidize to disulfides under physiological conditions.^46^ This drawback may offer a significant advantage for the inhibition of LecA, that is, multivalent interactions are among the most efficient ways invented by nature to enhance the lectin–carbohydrate interaction.^47,48^ Therefore, we speculated that the designed compounds will first inhibit LasB as thiols, while their excess at the site of infection inevitably dimerizes to give divalent galactosides with an enhancement in LecA binding affinity.

### Synthesis of dual LecA/LasB inhibitors

**Scheme 1:**
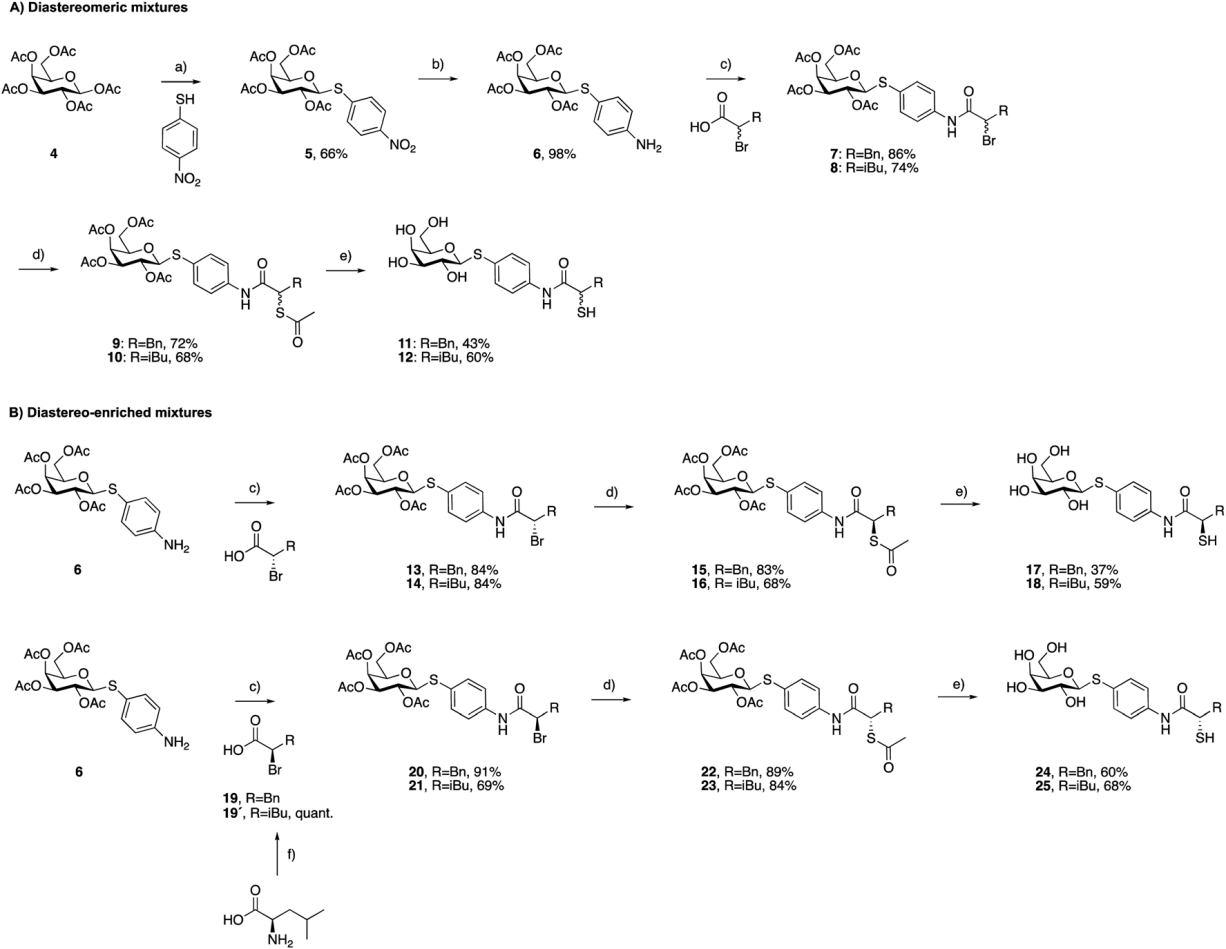
Synthesis of LecA/LasB dual inhibitors. (a) TfOH, MS 3 Å, DCM, 0 °C, 30 min; (b) H_2_, Pd/C, DCM, r.t., 18 h; (c) EDC*x*HCl, DCM, r.t., 4 h; (d) KSAc, acetone, r.t., 2 h; (e) NaOMe, MeOH, r.t., 45 min; (f) NaNO_2_, 48% HBr, H_2_O, 0 °C to r.t, 3 h.

As a first step, we synthesized glycomimetic thiols 11 and 12 in the form of diastereomeric mixtures (Scheme 1A). 4-Nitrothiophenol was glycosylated with β-d-galactose pentaacetate (4) using triflic acid as a catalyst to give β-thioglycoside 5 in 66% yield. The reduction of the nitro group to aniline derivative 6 using Pd/C-catalyzed hydrogenation was followed by amide coupling with racemic 2-bromo-4-methylpentanoic acid or 2-bromo-3-phenylpropanoic acid, which afforded compounds 7 and 8 in 86% and 74% yield, respectively. Bromides 7 and 8 were converted into thioacetates 9 and 10*via* nucleophilic substitution with potassium thioacetate in good yields. The subsequent deprotection under Zemplén conditions with sodium methoxide in methanol furnished thiols 11 and 12 in 43% and 60% yield, respectively.

To investigate whether stereochemistry has an impact on the activity, we synthesized two pairs of diastereomers, utilizing enantiomerically pure starting materials (Scheme 1B). In the case of compounds 17, 18 and 24, the corresponding α-bromo carboxylic acids with inverse stereochemistry were used. In the case of 25, the synthesis started from d-leucine affording (*R*)-2-bromo-4-methylpentanoic acid (19′) in quantitative yield, based on the work of Izumiya and Nagamatsu.^49–51^ The four derivatives were obtained following the procedures described for the diastereomeric mixtures (Scheme 1).

For the α-benzyl derivatives 17 and 24, the high degree of diastereomeric purity has been conserved during the three-step reaction cascade, as evidenced by the comparison of the NMR signals corresponding to the anomeric carbon atoms in the ^13^C NMR spectra of the diastereomeric mixture 11 and the separate diastereomers. Diastereomeric mixture 11 showed two signals at 88.27 and 88.23 ppm, corresponding to the anomeric carbon atoms of the two diastereomers, while 17 and 24 demonstrated one single signal each with a 0.04 ppm difference in chemical shifts (ESI†). The anomeric carbon atom in diastereomeric mixture 12 and in both isomers 18 and 25 appears as a single signal at 88.3 ppm, due to the smaller and/or more flexible isobutyl substituent (ESI†).

**Fig. 3:**
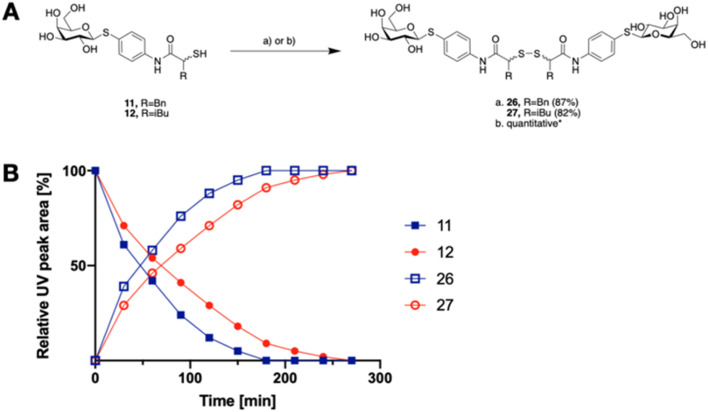
A) Oxidative dimerization of thiols 11 and 12 into disulfides 26 and 27 using (a) H_2_O_2_, DMSO/H_2_O or (b) *P. aeruginosa* culture supernatant (yield based on LC-MS); (B) dimerization kinetics of thiols 11 or 12 in the presence of *P. aeruginosa* culture supernatant into disulfides 26 or 27 analyzed by LC-MS.

The tendency of thiols to form disulfides in an oxidative environment suggests that our monovalent inhibitors will dimerize *in situ* over time, forming structures that can serve as divalent LecA inhibitors with enhanced potency against LecA. Disulfide formation in the presence of *P. aeruginosa* culture supernatant was studied for the two inhibitors 11 and 12 and analyzed by liquid chromatography-mass spectrometry (LC-MS) (Fig. 3B). Disulfides 26 and 27 were synthesized *via* oxidation with H_2_O_2_ in DMSO/H_2_O and used as reference compounds for the stability assay (Fig. 3A). For both, 26 (Bn) and 27 (*i*Bu), we observed two closely eluting substances with identical mass by LC-MS, suggesting that different diastereomers were formed. The separation of those two peaks of the benzylated derivative 26 using preparative HPLC gave 26a and 26b (*m*/*z* of 901.28, Fig. S1†).

In the presence of the bacterial culture supernatant of *P. aeruginosa*, the conversion of thiols 11 and 12 into the corresponding disulfides 26 and 27 was monitored at 37 °C and thiol half-lives of 48 min for 11 and 70 min for 12 were determined (Fig. 3B). The kinetics to convert the thiols to the corresponding disulfides allows 11 and 12 to first act as LecA and LasB dual inhibitors and to transform over time into more potent divalent inhibitors of LecA, 26 and 27 (Fig. 3).

### Activity against antivirulence targets LecA and LasB

We next evaluated the six synthesized thiol derivatives 11, 12, 17, 18, 24 and 25 for their inhibitory activity against both LecA and LasB. In addition, we determined the inhibitory activity of disulfides 26 and 27 on LecA but not on LasB as the essential thiol for coordination to the zinc ion is absent, leading to an expected loss in inhibitory activity.

**Fig. 4:**
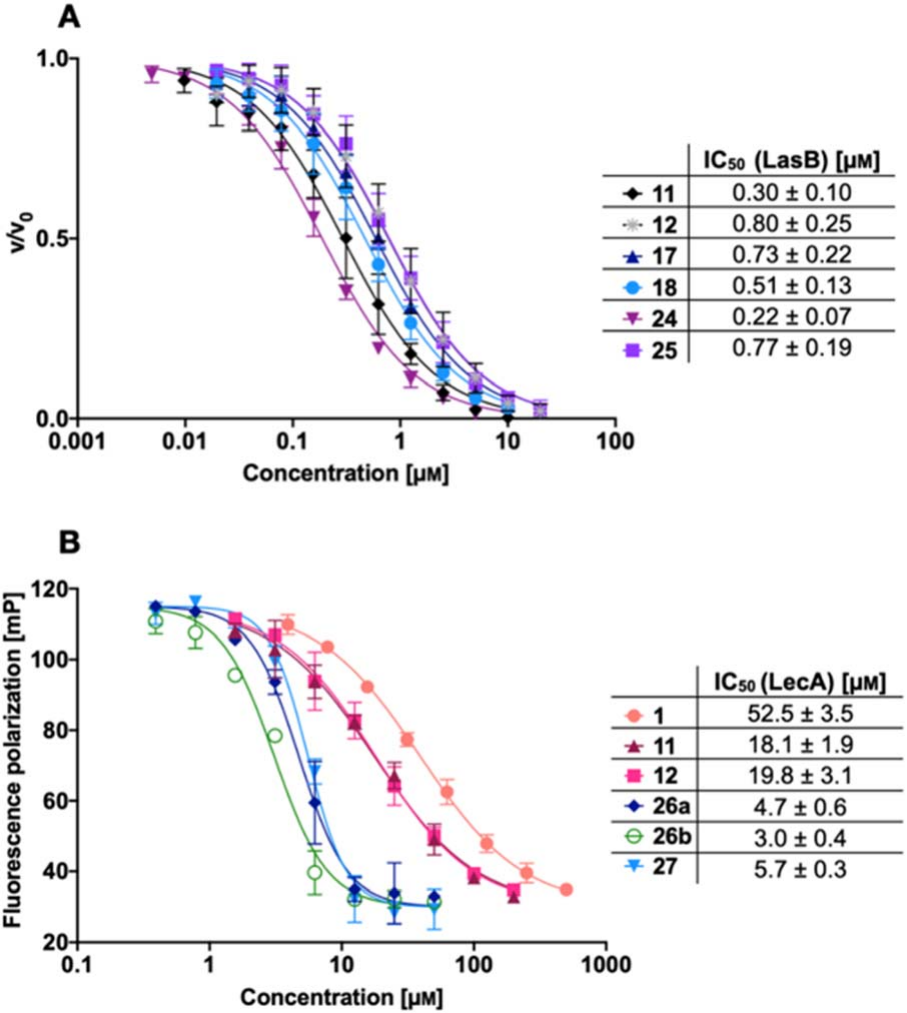
A) Evaluation of LecA/LasB dual inhibitors by a FRET-based *in vitro* LasB inhibition assay; (B) evaluation of monomeric LecA/LasB inhibitors 11 and 12 and divalent LecA inhibitors 26a, 26b and 27 and comparison to the control pNP-Gal (1) by a competitive binding assay for LecA based on fluorescence polarization. IC_50_ values were calculated from at least three independent experiments performed in triplicate.

The LasB activity of the thiol derivatives 11, 12, 17, 18, 24 and 25 was tested using a functional FRET-based *in vitro* proteolysis assay (Fig. 4A). Both α-isobutyl and α-benzyl derivatives demonstrated inhibitory activity against LasB in the same range as the previously observed activities of compounds 2 and 3 (IC_50_ = 0.40 and 1.2 μM, respectively).^37,41^ Interestingly, α-benzyl derivative 11 (IC_50_ = 0.30 μM) showed a four-fold improvement in activity compared to 3. α-Isobutyl derivative 12 (IC_50_ = 0.80 μM) proved to be somewhat less potent than the α-benzyl 11, and its diastereomer with (*R*)-configuration on the right-hand side of molecule 18 showed a slightly lower IC_50_ of 0.51 μM compared to its (*S*)-isomer (25, IC_50_ = 0.77 μM). On the other hand, the (*S*)-isomer 24 (IC_50_ = 0.22 μM) proved to be three-fold more potent compared to the (*R*)-isomer 17 (IC_50_ = 0.73 μM) among the α-benzylated series.

The four thiols 17, 18, 24 and 25 as well as three disulfides 26a, 26b and 27 were then evaluated for their activity against LecA using a competitive binding assay based on fluorescence polarization (Fig. 4B). The IC_50_ values obtained for LecA inhibition suggested that the addition of the LasB-inhibiting moiety has a positive effect on the affinity towards LecA, decreasing the IC_50_ more than two-fold, from 52.5 μM for 1 to 18.1 μM and 19.8 μM for 11 and 12, respectively. The substitution of the isobutyl residue with a benzyl group did not affect the affinity of disulfides in the LecA assay (IC_50_ = 4.7 μM and 3.0 μM for 26a and 26b, respectively, and IC_50_ = 5.7 μM for 27), but had an impact on the solubility of the corresponding disulfide. While isobutyl disulfide 27 showed a kinetic solubility >600 μM in 10 mm PBS with 2% DMSO at 37 °C, both benzyls 26a and 26b had a kinetic solubility of only 300 μM under the same conditions.

In this competitive binding assay, the observed affinity of the divalent LecA-inhibitors 26a, 26b and 27 increased more than three-fold compared to the corresponding thiols 11 and 12 (Fig. 4B). Considering the steep Hill slopes >2 of the fitted curves (Fig. 4B), we suspected that both divalent compounds approached the lower limit of the assay as observed before for inhibitors with significantly increased binding strength compared to the fluorescent primary ligand.^31,33^

**Fig. 5:**
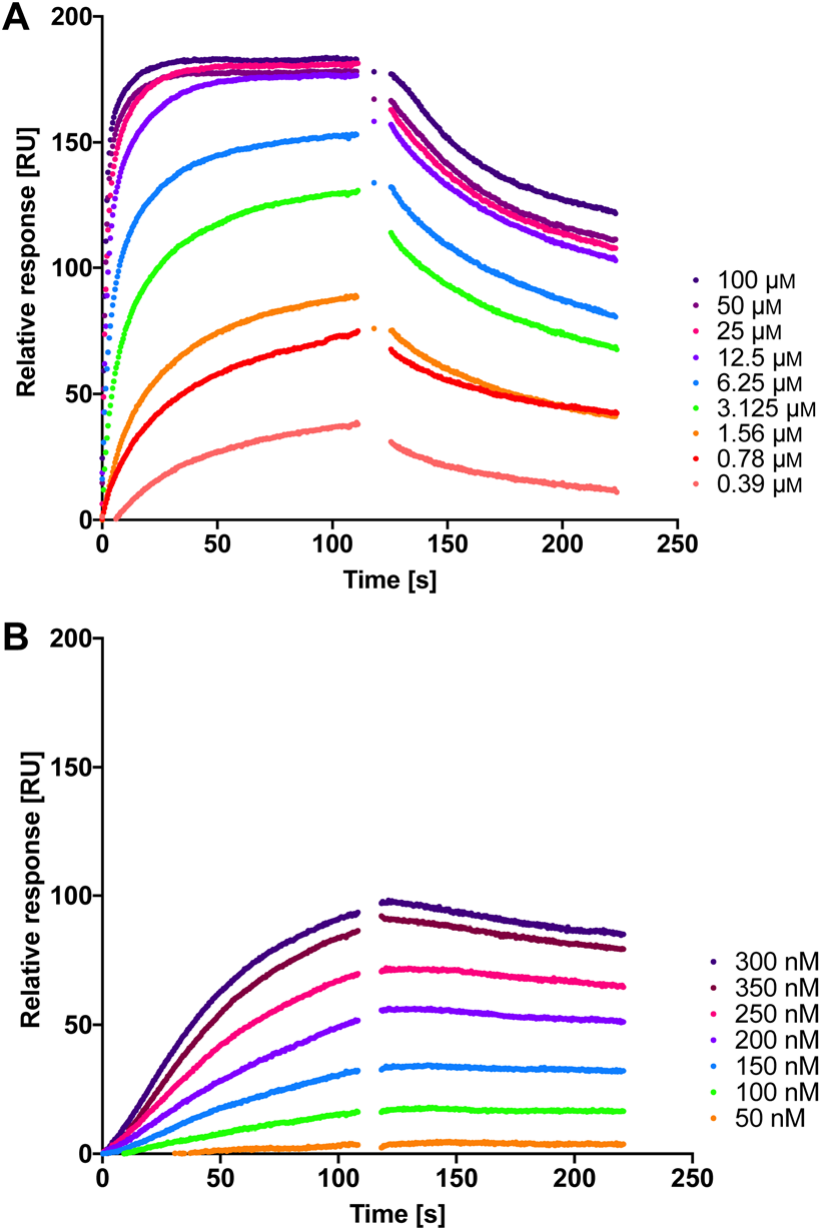
The interaction of dual inhibitors thiol 17 (A) and disulfide 26a (B) with LecA studied using SPR.

Therefore, we measured the LecA affinity of thiol derivatives 17, 18, 24 and 25 and disulfides 26a, 26b and 27 using surface plasmon resonance (SPR) with LecA covalently immobilized on a sensor chip *via* amide coupling (Fig. 5 and Table 1). The data demonstrate a significant increase in the affinity of the divalent compounds for LecA. Based on the fitting of the kinetic binding curves, divalent compounds displayed an up to 200-fold increase in activity (*K*_D_ = 7.4 nM and 6.6 nM for 26a and 26b, respectively, and *K*_D_ = 4.5 nM for 27) compared to their monovalent thiol analogues (*K*_D_ values = 1300 nM, 630 nM, 840 nM and 1000 nM for 17, 18, 24 and 25, respectively), shifting *K*_D_ values from the low-micromolar to the single-digit nanomolar range (Fig. 5 and S3†). Thiols 17, 18, 24 and 25 reached equilibrium binding within 60 s of interaction with immobilized LecA, followed by their dissociation with moderate off-rates. In contrast, the disulfides demonstrated very slow association rates with an approximately 10-fold increase of *k*_a_ compared to the value for 1 (Table 1).^52^ Due to the very small off-rates for 26a, 26b and 27, these values have been calculated based on the response at 350 nm of the injected compound and monitoring their dissociation for 30 min (Fig. S2†). The observed very tight binding to immobilized LecA required optimization of the protein surface regeneration procedure. Effective conditions were identified as one injection of 50 mm isopropyl β-d-thiogalactoside in the running buffer, followed by one injection of 20 mm EDTA disodium salt in water. The regenerated chip surface was controlled using an injection of 1 to ensure that the activity of LecA was maintained.

**Table 1:** Kinetic analysis of LecA/LasB inhibitors binding to immobilized LecA using SPR^a^

Compound	*k*_a_ [×10^4^ M^−1^ s^−1^]	*k*_d_ [×10^−4^ s^−1^]	*K*_D_ [nM]
1	0.40 ± 0.05	1000 ± 330	21 000 ± 4600
17	0.71 ± 0.14	86 ± 20	1300 ± 540
18	1.41 ± 0.47	82 ± 11	630 ± 290
24	0.88 ± 0.23	72 ± 5	840 ± 160
25	0.90 ± 0.31	85 ± 2	1000 ± 590
26a	3.8 ± 0.8	2.8 ± 0.3	7.4 ± 1.4
26b	4.6 ± 0.7	3.0 ± 1.2	6.6 ± 2.4
27	5.2 ± 0.6	2.2 ± 0.3	4.5 ± 0.4

a Mean values and standard deviations are from at least three independent experiments.

### Selectivity and toxicity profile of thiols 11 and 12

Given that the novel inhibitors contain a free thiol as a strong zinc binding group, we analyzed the selectivity of the diastereomeric mixtures 11 and 12 on six human matrix metalloproteases (MMPs) as putative off-targets.^53^ The data obtained demonstrate selectivity of both compounds for the two intended targets over all six off-targets tested since less than 20% inhibition was observed at 100 μM (Table S1†). Furthermore, we tested the impact of 11 and 12 on bacterial viability to ensure that these compounds are antivirulence agents and not antibiotics. No antimicrobial activity was detected up to 100 μM (Table S2†). Finally, we evaluated the cytotoxicity of compounds 11 and 12 against three human cell lines, HepG2, HEK293, and A549, revealing no detectable cytotoxicity at 100 μM (Table S3†).

Having established the inhibitors' high potency on both antivirulence targets and outstanding toxicity and selectivity properties, we set off to determine the modes of interaction with their targets LecA and LasB at the molecular level. Unfortunately, crystals could not be obtained for LecA with the observable electron density of thiols or the very potent disulfides. The crystal structures of multivalent ligands with multivalent LecA are intrinsically difficult to obtain and only one example has been reported to date, which also displays only incomplete electron density of the ligand.^54^

### Co-crystallization of thiol 11 with LasB

To get better insights into the binding mode and to understand the potency of the new inhibitors, we performed co-crystallization experiments with LasB, using the diastereomeric mixture of the α-benzylated analog 11.

**Fig. 6:**
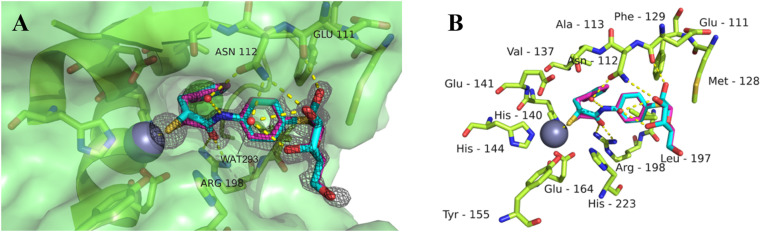
Crystal structure of 11 in complex with LasB (PDB: 7Z68). (A) Cartoon representation of LasB with a transparent surface (green) and ligated 11 (cyan (*R*), pink (*S*)). The amino acids of LasB forming the binding site are represented as sticks. The gray isomesh represents the polder map of 11 contoured at 3*σ*. (B) Stick representation of the LasB binding site with bound 11. Polar interactions between LasB and 11 are highlighted by dashed lines (color code: oxygen = red, nitrogen = blue, sulfur = yellow, and red spheres = water).

The LasB–11 complex crystallized in the space group *P*12_1_1, and crystals diffracted to 1.5 Å resolution (Fig. 6 and Table S4†). The obtained electron density of the ligand in the active site of LasB suggests that the enzyme accommodates both (*R*)- and (*S*)-isomers of compound 11. These data therefore explain why there is no stronger difference in the activities of the two isomers in the *in vitro* LasB assay ((*R*)-isomer 17 (IC_50_ = 0.73 μM) and (*S*)-isomer 24 (IC_50_ = 0.22 μM)).

As observed for previous crystal structures of thiols with LasB,^37,46^ also here the thiol of 11 displaces the water molecule in the tetrahedral coordination sphere of the zinc ion in the binding site, leading to a sulfur–zinc distance of 2.3 Å. The carbonyl oxygen of 11 forms a hydrogen bond with the side chain of Arg198 (3.1 Å) in the active site, stabilizing the binding of the compound in the core region. The benzyl ring is placed in the lipophilic S2′ pocket, thereby increasing the binding affinity *via* hydrophobic interactions. The galactose moiety is further stabilized by hydrogen bonds between its 2-hydroxy group (2-OH) and the backbone carbonyl of Glu111, two water-mediated hydrogen bonds *via* the same water (WAT293) between galactose 3-OH and 4-OH and the side chain of Asn112 and a second water-mediated hydrogen bond of 3-OH and the same side chain of Asn112. Thus, the side chain of Asn112 is heavily involved in coordinating through its amide-NH_2_ to the ligand's carbohydrate moiety with two water-mediated hydrogen bonds and its amide-oxygen forming a hydrogen bond with the ligand’s amide NH. An interesting observation is the unexpected folding of the aglycon, which leads to an intramolecular spatial proximity of hydroxyl 4-OH with its phenyl aglycon. Distances as close as 4.5 Å between 4-OH oxygen and the aryl carbon atom connected to the sulfur indicate attractive intramolecular ROH-π bonding.

### *In vitro* antivirulence evaluation of 1, 2 and 12 on A549 cells

**Fig. 7:**
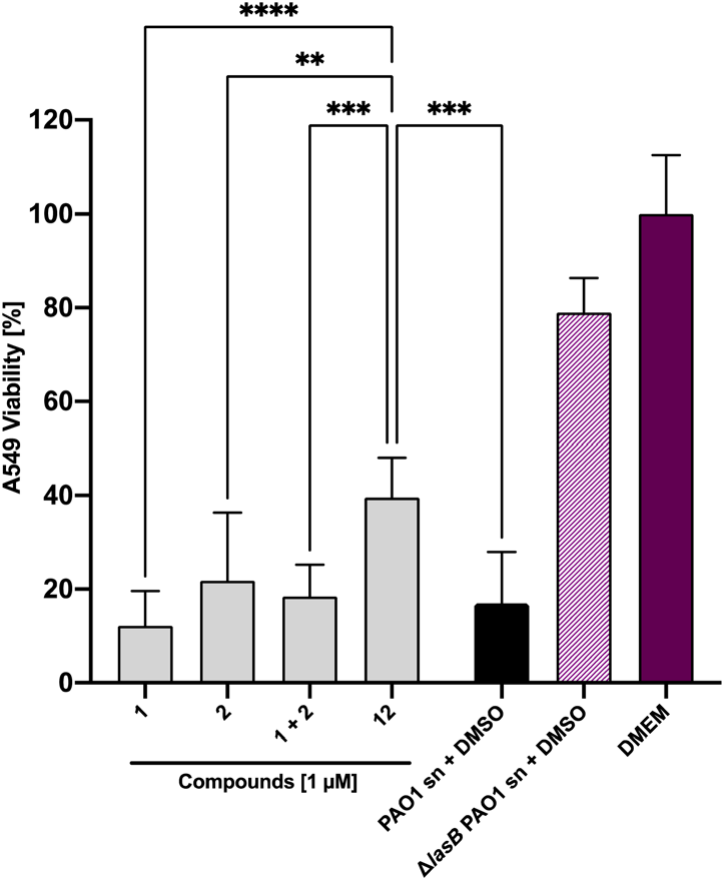
Inhibition of the LasB-dependent cytotoxicity of *P. aeruginosa* PAO1 culture supernatant on human A549 cells. Effect of PAO1 supernatant in the absence (black bar) or presence (grey) of compounds 1, 2, 1 + 2 or 12. The culture supernatant of the *lasB* knockout mutant (Δ*lasB* PAO1) was applied as a positive control (light purple). The viability of cells that were untreated (DMEM) served as a negative control and are shown in dark violet. The graphs represent the means of three independent experiments ± SD. One-way ANOVA statistical analysis was performed following Dunnett's multiple comparisons test, comparing the mean value of each concentration to the mean value of PAO1 without any treatment with compounds (***p ≤ 0.001, **p ≤ 0.01, and *p ≤ 0.05).

Next, we set out to analyze the ability of the dual inhibitor 12 and the individual treatment or combination of galactoside 1 and thiol 2 to reduce LasB-dependent cytotoxicity from *P. aeruginosa* PAO1 culture supernatant on A549 cells. None of the investigated compounds influenced A549 cell viability at 100 μM in the absence of bacterial culture supernatant (Fig. S4†). When A549 cells were exposed to *P. aeruginosa* PAO1 culture supernatant, both 100 μM and 10 μM of 12 led to an increase in cell viability compared to the combination of the individual LasB (2) and LecA (1) inhibitors at the corresponding concentrations (Fig. S5†). At 100 μM of 12, the cytotoxicity of PAO1 culture supernatant reached that of the LasB-deficient strain, which served as the control (Fig. S5†). At 1 μM, only the dual inhibitor 12 had a statistically significant beneficial effect on cell viability, whereas neither 1 nor 2 nor their combination had any effect (Fig. 7 and S5†). These data demonstrate the enhanced potency of the dual inhibitor against the secreted *P. aeruginosa* virulence factors.

### Evaluation of the dual inhibitors in LecA adhesion to A549 cells

**Fig. 8:**
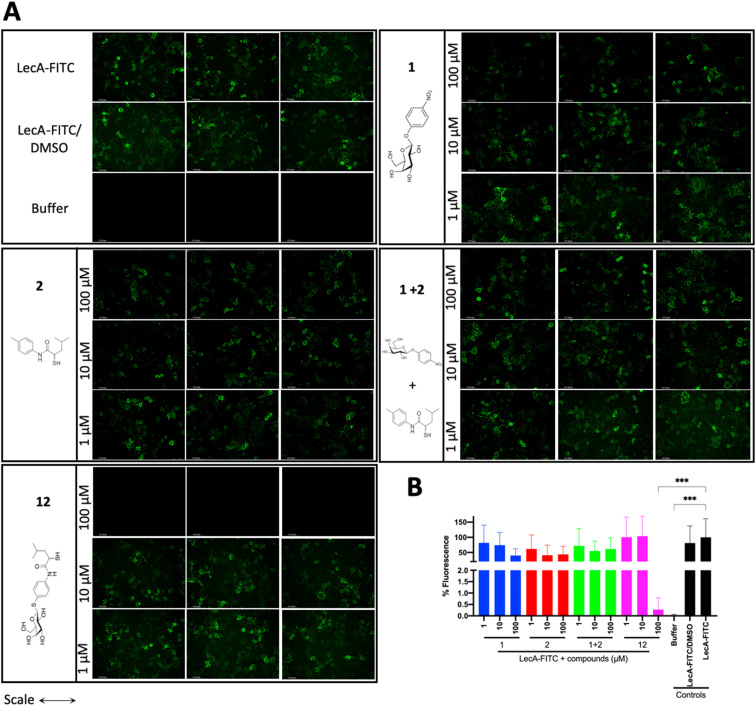
Analysis of the inhibitory activity of 1, 2, a combination of 1 + 2 and 12 on the adhesion of fluorescein-labelled LecA to human A549 cells by fluorescence microscopy. (A) Three representative fluorescence images of one biological replicate of LecA-FITC bound to A549 cells in the presence of the different compounds and under different concentrations (scale bar corresponds to 250 μM). (B) Quantification of mean image fluorescence intensities with the averages and standard deviations for 3 biological replicates ((A), S7A and B†). Intensities are normalized with LecA-FITC in the absence of inhibitors to 100% and in the absence of FITC-LecA to 0%. One-way ANOVA statistical analysis was performed following Dunnett’s multiple comparisons test, comparing the mean value of each condition to the mean value of the LecA-FITC positive control (***p ≤ 0.001, all other data have no statistical significance compared to the LecA-FITC positive control).

LecA-mediated cell adhesion is crucial for the initial infection and host-cell invasion of *P. aeruginosa*. Therefore, compounds 1 and 2, a combination of 1+2 and the dual inhibitor 12 were assessed for their ability to inhibit LecA-binding to human A549 cells. 10 μM of LecA-FITC and different concentrations of the respective compounds were incubated at 4 °C for 30 min with confluent A549 cells. After extensive washing, the analysis of the cells *via* fluorescence microscopy revealed that only the dual inhibitor 12 at 100 μM significantly inhibited the binding of LecA-FITC to A549 cells to the background level. No visible fluorescence signal was observed, which was comparable to the negative control of A549 cells in the absence of labeled LecA (Fig. 8). All other compounds and concentrations tested did not show a noticeable reduction in the fluorescence signal compared to the positive control consisting of A549 cells and LecA-FITC (Fig. 8 and S7†). The observation that the dual inhibitor showed high inhibition of LecA cell adhesion, while compounds 1 and 2 alone or in combination failed to do so, further highlights the superiority of 12.

## Conclusions

We report the first dual inhibitors of the major virulence factors LecA and LasB from the WHO priority I pathogen *P. aeruginosa*. Quite remarkably, our dual inhibitors displayed improved inhibitory activity and affinity for both targets down to 220 nM for LasB and 18 μM for LecA, compared to the individual predecessors of 400 nM and 53 μM, respectively. Implied by design, the divalent disulfide derivatives are formed *in situ*, which resulted in a 200-fold increase reaching single-digit nanomolar LecA activity determined using SPR (*K*_D_s 4.5–7.4 nM). We anticipate that the observed conversion rates of thiols into disulfides in the presence of the bacterial culture supernatant of *P. aeruginosa* are beneficial to ensure the initial saturation of LasB with the thiol and allow the subsequent inhibition of LecA with the potent bivalent disulfides. The crystal structure of the dual inhibitor 11 in complex with LasB confirmed the interaction at the atomic level. To further assess the potential of these merged inhibitors as candidates for therapeutic applications at this early stage, we evaluated their selectivity and toxicity profiles. Importantly, the compounds did not show any inhibition of a panel of six human MMPs as potential off-targets and cytotoxicity was not observed. Finally, we demonstrated a reduction of the cytotoxicity of *P. aeruginosa* culture supernatant by the dual inhibitor 12 on A549 cells *in vitro*, which primarily originates from secreted LasB, as well as an efficient inhibition of LecA adhesion to A549 cells. Both inhibitory effects were observed only for the dual inhibitor 12, but were absent for single or combination treatments with LecA- or LasB inhibitors 1 and 2, respectively.

Disarming highly pathogenic *P. aeruginosa* by interfering with its major pathogenicity factors offers a promising new option for therapeutics. Future research will focus on infection models using more complex *in vivo* systems, to support the importance of the presented dual inhibitors and their translation into practical applications.

## Experimental section

Experimental details, materials, methods, chemical syntheses and transcripts of ^1^H and ^13^C NMR spectra can be found in the ESI.†

## Data availability

The datasets supporting this article have been uploaded as part of the ESI.† The crystal structure dataset has been deposited at the Protein Database under code 7Z68, https://www.rcsb.org/structure/7Z68.

## Author contributions

O. M. and J. K. contributed equally. All authors discussed the results and commented on the manuscript. Design of the study: O. M., J. K., S. Y., A. T., A. K. H. H.; project management A. T., A. K. H. H.; acquisition of funding: A. T., A. K. H. H.; project supervision: A. T., A. K. H. H.; design and chemical synthesis: O. M., J. K., S. Y.; evaluation of oxidative dimerization of thiols by LC-MS: O. M.; evaluation of LasB activity by *in vitro* inhibition assay: J. K.; evaluation of LecA binding by fluorescence polarization assay and SPR: O. M.; cytotoxicity and selectivity profile: J. H.; X-ray: A. K.; *in vitro* antivirulence evaluation: A. A., R. S.; LecA adhesion to A549 cells *in vitro*: R. S., M. F.; writing original draft: O. M., J. K.; writing – review and editing: O. M., J. K., A. T., A. K. H. H., with a contribution of all authors.

## Conflicts of interest

The authors declare no competing financial interest.

## Supplementary Material

SC-015-D4SC02703E-s001
