# Supplementary material for: Dual inhibitors of Pseudomonas aeruginosa virulence factors LecA and LasB
Source: Chem Sci. 2024 Jul 22;15(33):13333–42. doi: 10.1039/d4sc02703e (PMC11339798; doi:10.1039/d4sc02703e)
Supplement: SC-015-D4SC02703E-s001 [file SC-015-D4SC02703E-s001.pdf]

Electronic Supplementary Material (ESI) for Chemical Science.
This journal is © The Royal Society of Chemistry 2024

Supporting information

Dual inhibitors of *Pseudomonas aeruginosa* virulence factors LasB and LecA

Olga Metelkina[▽a,b,c], Jelena Konstantinović[▽a], Andreas Klein[a,d], Roya Shafiei[a,d], Mario Fares [a,b,c], Alaa Alhayek,[a] Samir Yahiaoui[a], Walid A. M. Elgaher,[a] Jörg Haupenthal[a], Alexander Titz*[a,b,c], Anna K. H. Hirsch*[a,b,d]

[a]Helmholtz Institute for Pharmaceutical Research Saarland (HIPS) – Helmholtz Centre for Infection Research (HZI), 66123 Saarbrücken, Germany

[b]Deutsches Zentrum für Infektionsforschung (DZIF), Standort Hannover-Braunschweig, 38124 Braunschweig, Germany

[c]Saarland University, Department of Chemistry, 66123 Saarbrücken, Germany

[d]Saarland University, Department of Pharmacy, 66123 Saarbrücken, Germany

[▽]These authors contributed equally.

*corresponding authors: anna.hirsch@helmholtz-hips.de, alexander.titz@helmholtz-hips.de

General experimental details.

Commercial chemicals and solvents were used without further purification. Procedures were not optimized regarding yield. Thin layer chromatography (TLC) was performed using TLC Silica gel 60 F_{254} sheets (Merck KGaA, Darmstadt, Germany) and developed under UV light (254 nm) and using a cerium molybdate stain (0.02 M solution of $(NH_4)Ce(SO_4)_4 \cdot 2H_2O$ and $(NH_4)_6Mo_7O_{24} \cdot 4H_2O$ in aqueous 10% H_2SO_4) or a permanganate stain (3 g of $KMnO_4$, 20 g of K_2CO_3 in 5 mL of 5% NaOH and 300 mL of water) with heating. Medium pressure liquid chromatography (MPLC) was performed on a Combiflash Rf200 (Teledyne Isco) system using normal phase silica gel columns (60 Å, 400 mesh particle size, Fluka) or reversed-phase silica gel columns Chromabond Flash C_{18} ec (Macherey-Nagel, Düren, Germany). Preparative high-pressure liquid chromatography (HPLC) was performed on Waters 2545 Binary Gradient Module with a Waters 2489 UV/Vis detector using a RP-18 column (Nucleodur C18 Gravity SB, 5 µm, 250x21 mm from Macherey-Nagel, Germany) and DionexUltiMate 3000 UHPLC+ focused (Thermo Scientific, United States) using a reversed-phase C18 column (Hypersil gold, 5 µm, 250x10 mm from Macherey-Nagel, Germany). Analytical HPLC-MS was performed using: a) Thermo Dionex Ultimate 3000 HPLC (Thermo Scientific, United States) coupled to a Bruker amaZon SL mass spectrometer, with UV detection at 254 nm using a C18 column (Nucleoshell RP18plus, 2.7 µm, 100x2 mm from Macherey-Nagel, Germany) as stationary phase; b) ESI quadrupole MS (MSQ Plus or ISQ EC, Thermo Fisher Scientific, Dreieich, Germany) using C18 column (Hypersil gold, 3 µm, 100x2 mm from Thermo Scientific, United States) and c) Ultimate 3000 HPLC (Thermo Fisher) coupled to a TSQ Quantum Access Max triple quadrupole mass spectrometer (Thermo Fisher) using C18 column (Hypersil gold, 1.9 µm, 100x2 mm from Thermo Scientific, United States). Purity of the final compounds was determined by LC-MS using the UV trace recorded at a wavelength of 254 nm and found to be >95%. High resolution mass spectrometry (HRMS) was performed on an Ultimate 3000 UPLC system coupled to a Q Exactive Focus Orbitrap system with HESI source (Thermo Fisher, Dreieich, Germany). The UPLC was operated with a C18 column (EC 150/2 Nucleodur C18 Pyramid, 3 µm from Macherey-Nagel, Germany). ^{1}H- and ^{13}C-NMR spectra were recorded on a Bruker Avance III 500 UltraShield spectrometer at 500 MHz and 126 MHz, respectively. Chemical shifts (δ) are given in ppm and were calibrated on residual solvent peaks: $CHCl_3$-d_1 (^{1}H-NMR δ = 7.26 ppm, ^{13}C-NMR δ = 77.0 ppm), MeOH-d_4 (^{1}H-NMR δ = 3.31 ppm, ^{13}C - NMR δ = 49.0 ppm), DMSO-d_6 (^{1}H-NMR δ = 2.50 ppm, ^{13}C-NMR δ = 39.51 ppm). Deuterated solvents were purchased from Eurisotop (Saarbrücken, Germany). Multiplicities are specified as s = singlet, bs = broad singlet, d = doublet, dd = doublet of doublets, t = triplet, td = triplet

of doublets, q = quartet, m = multiplet. The spectra were assigned with the help of ^{1}H, ^{1}H-COSY and ^{1}H,^{13}C-HSQC experiments.

Expression and purification of LasB and LecA

LecA was expressed recombinantly using *E. coli* pET25-pail, whereas LasB was isolated from *P. aeruginosa* PA14 culture supernatant as described previously.[1,2]

LecA labeling with fluorescein was performed in analogy to Sommer et al. [3] and Beshr et al.[4] To this end fluorescein isothiocyanate (FITC) was freshly dissolved in carbonate-bicarbonate buffer with pH of 9.3 at a concentration of 3 mg mL^{-1}. Subsequently, 66 µL of this FITC solution was combined with 1.4 mL of LecA at a concentration of 60 µM in carbonate-bicarbonate buffer, pH 9.3. The resulting mixture was then incubated at 25 °C with shaking at 500 rpm for 1 hour. Then, extensive washing with PBS/Ca^{2+} in a 5 kDa molecular weight cutoff centrifugal concentrator was performed to remove excess dye and buffer the labelled protein.

In vitro inhibition assay for LasB

The in vitro inhibition assay was performed as described previously,[2] using purified LasB (final concentration 0.3 nM), the fluorogenic substrate 2-aminobenzoyl-Ala-Gly-Leu-Ala-4-nitrobenzylamide (purchased from Peptides International (Louisville, KY, USA) or Vivitide, LLC (Gardner, MA, USA), final concentration 150 µM)) in assay buffer (50 mM Tris, pH 7.2, 2.5 mM $CaCl_2$, 0.075% Pluronic F-127, 5% DMSO). Fluorescence was measured using a CLARIOstar plate reader (BMG Labtech, Ortenberg, Germany). IC_{50} values were calculated using GraphPad Prism 8 (GraphPad Software, San Diego, CA, USA) from at least three independent experiments performed in duplicate.

Competitive binding assay for LecA

The procedure for the fluorescence polarization-based competitive binding assay for LecA was performed as reported by Joachim et al. with modifications.[1] The assay was performed in TBS/Ca^{2+} buffer (20 mM Tris, 137 mM NaCl, 2.6 mM KCl at pH 7.4 supplemented with 1 mM $CaCl_2$ and contained 5% DMSO). The final protein concentration in the assay was 5 µM for LecA. Fluorescence intensities were recorded on a PheraStar FS plate reader (BMG Labtech GmbH, Germany) and polarization was calculated using MARS Data Analysis (BMG Labtech). IC_{50} values were obtained from a four-parameter curve-fitting procedure. Averages and standard deviations were calculated from at least three independent experiments of technical triplicates each.

Surface plasmon resonance direct LecA binding assay

SPR experiments were performed on a Reichert 2-channel SPR SR7500DC (Reichert Technologies Life Sciences, Buffalo, NY, USA) at 25 °C. For LecA immobilization, the surface of a HC1000M sensor chip (XanTec bioanalytics GmbH, Düsseldorf, Germany) was preequilibrated with borate buffer (1 M NaCl, 0.1 M sodium borate pH 9.0), followed by activation of the polycarboxylate hydrogel with 0.2 M *N*-hydroxysuccinimide (NHS) and 0.4 M 1-ethyl-3-(3-dimethylaminopropyl)carbodiimide hydrochloride (EDC·HCl) in MilliQ water in both channels for 240 s (flow rate 10 µL/min) until the binding response reached 1200 RU. Then, LecA (100 µg/mL) in 10 mM sodium acetate pH 3.6 was injected on the activated chip surface of one channel (injection time 420 s, flow rate 10 µL/min) and 6000 RU of LecA were immobilized. Unreacted NHS esters in both channels were blocked with 1 M ethanolamine hydrochloride in MilliQ water pH 8.5 (injection time 360 s, flow rate 10 µL/min).

Test compound stock solutions were prepared in DMSO (20 mM stocks for monovalent LecA inhibitors, 20 µM stocks for divalent LecA inhibitors) and were subsequently diluted in 10 mM phosphate buffer (pH 7.4, 2.7 mM KCl, 137 mM NaCl, 100 µM $CaCl_2$) to obtain solutions with 2% DMSO.

Monovalent inhibitors **17**, **18**, **24** and **25** were injected for 120 s (flow rate 50 µL/min) at concentrations ranging from 0.39–100 µM. Divalent inhibitors **26a**, **26b** and **27** were injected for 120 s (flow rate 50 µL/min) at concentrations 50–350 nM. The chip surface was regenerated after each inhibitor injection by one injection of 50 mM IPTG in running buffer (10 mM phosphate buffer, pH 7.4, 2.7 mM KCl, 137 mM NaCl, 100 µM $CaCl_2$, 2% DMSO) followed by one injection 20 mM EDTA disodium salt in MilliQ water, and then followed by three injections of the running buffer (injection time 120 s, flow rate 50 µL/min). 100 µM pNP-Gal (**1**) in running buffer was injected before each analyte to monitor and ensure chip regeneration and protein activity.

Affinity/equilibrium analysis was performed for **1** on the same chip. A stock solution of **1** (10 mM in PBS/$CaCl_2$ buffer) was prepared, then diluted to the required concentrations in running buffer and injected at 1.56–400 µM (injection time 60 s, flow rate 50 µL/min) without regeneration steps. Data analysis was performed using TraceDrawer software (Ridgeview Instruments) using a 1:1 binding model to fit the experimental data. Spikes at the end of the injections were removed in TraceDrawer during the data analysis.

Disulfide formation assay

The stability of two thiols **11** and **12** and their ability to form the corresponding disulfides was analyzed by HPLC-MS. Chemically synthesized disulfides **26** and **27** were used as references for the stability assay. 50 mM DMSO stocks of thiols **11** and **12** were diluted 40-fold with *P. aeruginosa* PA14 overnight LB medium culture supernatant (25% v/v, sterile filtered through 0.22 µm filters in 10 mM phosphate buffer, pH 7.4, 2.7 mM KCl, 137 mM NaCl, 100 µM $CaCl_2$) at 37 °C. Samples were drawn over 4.5 hours and analyzed by LC-MS using a RP-18 column 100/2 Nucleoshell RP18plus, 2.7 µm from Macherey-Nagel, Germany (H_2O/MeCN + 0.1% formic acid, 15 – 55% MeCN, retention time (t_R) 5.8 min (**11** or **12)**, 6.1 min and 6.3 min (disulfide products **26** or **27**) on a Thermo Dionex Ultimate 3000 HPLC coupled to a Bruker amaZon SL mass spectrometer, with UV detection at 254 nm). Thiol half-lives were determined by analysis of the obtained graphs and values of 80 min for **11** and 46 min for **12** were obtained (Figure 2).

Selectivity and toxicity assays

Human MMPs -1, -2, -3, -7, -8 and -14 along with the SensoLyte 520 Generic MMP Activity Kit*Fluorimetric* were purchased from AnaSpec (Fremont, CA, USA). The inhibition assay was performed according to the guidelines of the manufacturer and as described recently.[5]
Cytotoxicity on human liver (HepG2), kidney (HEK293) and lung (A549) cells were performed using MTT assay and measuring the optical density (OD) photometrically at 570 nm in a FLUOstar Omega plate reader (BMG labtech, Ortenberg, Germany) as described previously.[6]
Antimicrobial activity against *P. aeruginosa* PA14 was tested using ESCMID guidelines and details have been reported previously.[7]

***In vitro* evaluation of 12 and a combination of 1 and 2 using *P. aeruginosa* culture supernatant and A549 cells in a cell viability assay**

The A549 cell line, derived from human lung adenocarcinoma, was grown in Dulbecco's Modified Eagle Medium (DMEM) supplemented with 10% Fetal Bovine Serum (FBS) and 1% penicillin-streptomycin. The cells were cultured following standard protocols for cell maintenance.
To prepare the bacterial culture supernatant, individual flasks containing 15 mL of lysogeny broth (LB) culture medium were inoculated with PAO1 or PAO1 Δ*lasB* strains. Culture flasks were placed on a shaker and incubated at 37 °C and 180 rpm overnight for 18 hours. Following incubation, the bacterial cultures were subjected to centrifugation at 4 °C and 5000 rpm for

10 min, facilitating the separation of bacterial cells from the supernatant. The latter was filtered through a 0.22 µm sterile filter and stored at –80 °C until further use.
In a flat bottom 96-well plate (Corning™ Costar™), each well was seeded with 2.5–3.5*10^3 cells in 100 µL DMEM/FCS and then incubated at a temperature of 37 °C with 5% CO_2 for 24 hours. Then, compounds **12** and **1** combined with **2** in concentrations of 100 µM, 10 µM, and 1 µM were mixed with 10% (v/v) of culture supernatant of *P. aeruginosa* PAO1 (DSM 22644, ATCC 15692) in DMEM/FCS. To minimize the potential negative effect of DMSO, the compound was initially dissolved in 99.9% DMSO, and a final assay concentration of 0.5% DMSO was applied. Additionally, the cells were exposed to 10% (v/v) PAO1 Δ*lasB* culture supernatant to analyze the effect caused by LasB, while DMEM/FCS without any treatment served as a control. The plates were then incubated at 37 °C with 5% CO_2 for 24 hours before conducting the MTT assay.

MTT assay

To assess cell viability, the medium in the well plates was aspirated and a single wash was performed using 100 µL of phosphate buffered saline, pH 7.4 (PBS). A solution of 5 mg MTT (3-(4,5-dimethylthiazol-2-yl)-2,5-diphenyl tetrazolium bromide) per mL PBS was prepared and diluted to a concentration of 10% (v/v) in DMEM/FCS. Subsequently, 100 µL of this MTT solution was added to each well, and the plates were incubated at 37 °C for 3 hours with 5% CO_2. After incubation, the MTT solution was carefully removed, and the formazan crystals were dissolved by adding 150 µL of lysis solution (250 mL DMSO, 25 g SDS, and 1.25 mL AcOH) to each well. The plates were then incubated for an additional 30 min at 37 °C and then, the UV absorbance was measured using a PHERAstar microplate reader. The test samples were read at a wavelength of 550 nm, while the blank was read at 620 nm. At last, the data were statistically analyzed and presented graphically using GraphPad Prism 9 (Figure S4 and S5).

Evaluation of compounds 1, 2 and 12 in inhibiting LecA adhesion to A549 cells via fluorescence microscopy

The A549 cell line were cultured following the protocols for cell maintenance described above. In a flat-bottom cell culture black 96-well plate (Greiner Bio-One, #Cat 655090) each well was seeded with 1*10^4 cells in 100 µL DMEM/FCS and subsequently, incubated overnight at 37 °C with 5% CO_2. On the following day, concentrations of 100 µM, 10 µM, and 1 µM of the compounds **1**, **2**, the combination of **1** and **2**, or **12** were incubated for 30 min at room temperature with 10 µM FITC-labeled LecA. Each test sample comprises 100 µL solution of 10

μM FITC-labeled LecA in DMEM/FCS and varying concentrations of the test compound (ranging from 1 μM to 100 μM). This prepared mixture was then added after 30 min incubation at room temperature to the cell culture wells in duplicate. The compounds were initially dissolved in 99.9% DMSO, and a final assay concentration of 0.5% DMSO was used. Furthermore, the A549 cells were exposed to 10 μM FITC-labeled LecA both with or without DMSO to quantify staining efficacy of LecA on A549 cells and to ensure that DMSO did not have any negative effects. The plate was then incubated at 4 °C for 30 min and wells were washed 5 times with PBS prior to imaging via fluorescence microscopy.

Fluorescence image acquisition, processing, and quantification

Fluorescence images were captured using an inverted fluorescence microscope (Leica DMi8) equipped with a 20x objective (HC PL APO CS2 20x/0.75 IMM UV), an FITC filter cube (EX: 460-500 nm, DC: 505, EM: 512-542 nm) and a monochrome microscope camera (Leica DFC7000 GT). The Leica Application suite X (3.7.4.23463) software navigator was used to image the whole well at one plane. The same image settings were used for imaging all the conditions in each biological replicate.

From each of the three biological replicates, three representative images were selected, each originating from two technical duplicates. Background fluorescence was subtracted using the LasX image processing software with the slider value at 500. Background-subtracted images were then exported as TIFF files, lossless compression, and scale bar were ticked.

The fluorescence signal was quantified with ImageJ by selecting the whole background subtracted image and calculating its mean fluorescence intensity. Data were plotted and statistically analyzed using GraphPad Prism.

X-ray Crystallography of the complex of 11 with LasB.

LasB at a concentration of 3.6 mg/mL was mixed with the diastereomeric mixture of **11** to get a final concentration of 1 mM compound in a 10 mM Tris buffer containing 2 mM $CaCl_2$. Crystal-screens have been set up using the sitting drop method and commercially available PEGs and PEGs II screen solutions (NeXtal Biotechnologies, 6201 Trust Drive Holland, OH 43528). The crystal for the final data set was obtained from the PEG F6 condition, containing 0.2 M potassium nitrate and 20% (w/v) PEG 3350. For data collection, the crystals were cryoprotected with a final concentration of 25% glycerol. Diffraction data were collected at beamline ID30B (ESRF, Grenoble, France) at 100 K and a wavelength of 0.9763 Å. The data was processed using CCP4 Aimless,[8] and the structure was solved by molecular replacement using *P. aeruginosa* LasB (PDB ID 6FZX) without the ligand as a search model in PHASER. The

solution was rebuilt using AutoBuild. Refinement was done with phenix.refine (Phenix version 1.19.2-4158-000)[9] and manually with WinCOOT (0.9.6)[10]. Images were created using PyMOL (2.5.2)[11] (Figure S6).

Synthesis

General procedure A: Synthesis of *N*-aryl-2-bromo-2-alkyl(aryl)acetamide derivatives

2-Haloalkanoic acid (1.2 equiv.) and EDC·HCl (1.2 equiv.) were added to a solution of the corresponding aniline (1.0 equiv.) in DCM. The resultant mixture was stirred at r.t. for 4 h. The solution obtained was washed with 1 M HCl and saturated aqueous NaCl solution. The organic layer was dried over anhydrous Na_2SO_4, filtered and concentrated under reduced pressure to afford the crude product. The crude product was purified using column chromatography.

General procedure B: Synthesis of *N*-aryl-2-thioacetyl-2-alkyl(aryl)acetamide derivatives

N-Aryl-2-bromo-2-alkyl(aryl)acetamide derivative (1.0 equiv.) was dissolved in acetone, and potassium thioacetate (2.0 equiv.) was added to the solution. The resultant mixture was stirred at r.t. for 2 h. After concentration under vacuum, the resultant residue was diluted with H_2O and extracted with EtOAc. The organic layer was washed with saturated aqueous NaCl solution, dried over anhydrous Na_2SO_4, filtered and evaporated under reduced pressure. The crude residue was purified using column chromatography.

General procedure C: Synthesis of *N*-aryl-2-mercapto-2-alkyl(aryl)acetamide derivatives

N-aryl-2-thioacetyl-2-alkyl(aryl)acetamide derivatives were dissolved in MeOH under argon atmosphere and NaOMe (5.33 M in MeOH, 4.0–7.0 equiv.) was added to this solution. The reaction was stirred at r.t. for 45 min. pH was neutralized with Amberlite IR-120. After filtration and evaporation of the solvent, the product was obtained as pure material or purified using preparative HPLC.

General procedure D: Synthesis of disulfides

Thiols obtained as diastereomeric mixtures were dissolved in DMSO/H_2O 1:1 and 3% (v/v) H_2O_2 (2.5 equiv.) was added, and the reaction was stirred at r.t. for 4 h. The reaction mixture was diluted with ice-cold water and lyophilized. The product was purified by preparative HPLC.

***p*-Nitrophenyl 2,3,4,6-tetra-*O*-acetyl-β-D-thiogalactopyranoside (5)**.

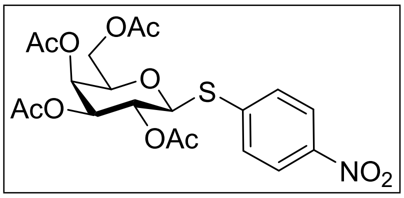

Penta-*O*-acetyl-β-D-galactose **4**[12] (2 g, 5.12 mmol) and *para*-nitro-thiophenol (1.59 g, 10.24 mmol) were dissolved in 20 mL dry dichloromethane with 200 mg 3 Å activated molecular sieves under nitrogen flow. The reaction mixture was cooled down to 0 °C on an ice bath, triflic acid (360 μL, 4.09 mmol) was added dropwise via a cannula and stirred for 30 min. The reaction was diluted with dichloromethane, filtered through celite and washed with sodium bicarbonate, water and saturated aqueous NaCl solution sequentially, then dried over anhydrous sodium sulfate. After filtration, the organic phase obtained was concentrated in vacuo and recrystallized twice from EtOAc to provide **5** as white crystals (1.65 g, 66%). The acquired ^{1}H and ^{13}C NMR spectra (500 MHz) were found in agreement with reported data of Escopy *et al.*[13]

***p*-Aminophenyl 2,3,4,6-tetra-*O*-acetyl-β-D-thiogalactopyranoside (6)**.

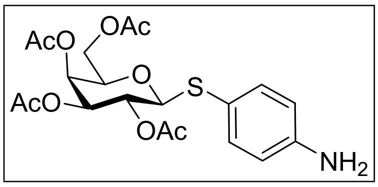

Compound **5** (900 mg, 1.86 mmol) was suspended in dry dichloromethane (40 mL), and palladium on activated charcoal (90 mg, 10% w/w) was added under a nitrogen atmosphere. The reaction flask was flushed with hydrogen and stirred at room temperature overnight under a hydrogen atmosphere. The reaction mixture was filtered through celite, and the solvent was removed *in vacuo.* The crude product was purified by normal phase MPLC (PhMe/EtOAc, 40–80% EtOAc). **6** was obtained as a pale pink solid (818 mg, 97%). The acquired ^{1}H and ^{13}C NMR spectra (500 MHz) were found in agreement with reported data of Casoni *et al.*[14]

(2*R*,3*S*,4*S*,5*R*,6*S*)-2-(Acetoxymethyl)-6-((4-(2-bromo-3-phenylpropanamido)phenyl)thio)tetrahydro-2*H*-pyran-3,4,5-triyl triacetate (7)

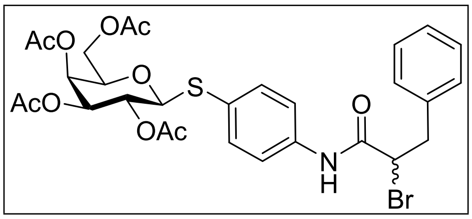

Compound **7** was synthesized according to the general procedure A, using 2-bromo-3-phenylpropanoic acid (72.5 mg, 0.32 mmol), compound **6** (120 mg, 0.26 mmol), EDC·HCl (60.4 mg, 0.32 mmol) and DCM (15 mL). The obtained crude product was purified using column chromatography (Hex/EtOAc = 6/4 to 1/1), to give compound **7** as a white solid (150.5 mg, 86%). ^{1}H NMR (500 MHz, $CDCl_3$) δ 7.85 (s, 1H), 7.53 – 7.49 (m, 2H), 7.47 – 7.43 (m, 2H), 7.35 – 7.25 (m, 5H), 5.40 (d, *J* = 3.2 Hz, 1H), 5.20 (td, *J* = 10.0, 2.2 Hz, 1H), 5.04 (dd, *J* = 9.9, 3.4 Hz, 1H), 4.64 (d, *J* = 9.9 Hz,

1H), 4.63 – 4.58 (m, 1H), 4.21 – 4.15 (m, 1H), 4.14 – 4.08 (m, 1H), 3.91 (t, *J* = 6.6 Hz, 1H), 3.61 (ddd, *J* = 14.3, 6.0, 2.1 Hz, 1H), 3.35 (ddd, *J* = 14.3, 7.5, 1.8 Hz, 1H), 2.11 (s, 3 H), 2.10 (s, 3 H), 2.05 (s, 3 H), 1.97 (s, 3 H). ^{13}C NMR (126 MHz, $CDCl_3$) δ 170.6, 170.4, 170.2, 169.6, 166.2, 137.4, 136.72, 136.69 (two signals at 136.7 due to diastereomeric mixture), 134.3, 129.6, 128.7, 128.1, 127.6, 120.4, 86.8, 74.6, 72.1, 67.34, 67.32, 61.7, 51.81, 51.76 (two signals at 51.8 due to diastereomeric mixture), 41.7, 21.0, 20.9, 20.8, 20.7. MS (ESI^+) m/z calculated for $[C_{29}H_{33}BrNO_{10}S]^+$ 666.10 $[M+H]^+$, found 665.97.

(2*R*,3*S*,4*S*,5*R*,6*S*)-2-(Acetoxymethyl)-6-((4-(2-bromo-4-methylpentanamido)phenyl)thio)tetrahydro-2*H*-pyran-3,4,5-triyl triacetate (8)

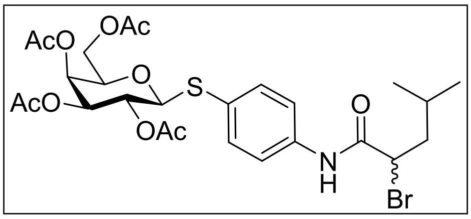

Compound **8** was synthesized according to the general procedure A, using 2-bromo-4-methylpentanoic acid (87.6 mg, 0.45 mmol), EDC·HCl (86.1 mg, 0.45 mmol), aniline **6** (170.5 mg, 0.37 mmol) and DCM (7.5 mL). The crude product obtained was purified using column chromatography (Hex/EtOAc = 1:1), to give compound **8** as a white solid (174.5 mg, 74%). ^{1}H NMR (500 MHz, $CDCl_3$) δ 8.04 (s, 1H), 7.51 (s, 4H), 5.41 (d, *J* = 2.6 Hz, 1H), 5.20 (t, *J* = 9.9 Hz, 1H), 5.03 (dd, *J* = 10.0, 3.3 Hz, 1H), 4.63 (d, *J* = 10.0 Hz, 1H), 4.43 (dd, *J* = 9.4, 5.5 Hz, 1H), 4.21 – 4.16 (m, 1H), 4.14 – 4.09 (m, 1H), 3.91 (t, *J* = 6.6 Hz, 1H), 2.12 (s, 3H), 2.10 (s, 3H), 2.05 (s, 3H), 2.03 – 1.95 (m, 2H), 1.97 (s, 3H), 1.95 – 1.85 (m, 1H), 1.00 (d, *J* = 6.6 Hz, 3H), 0.95 (d, *J* = 6.6 Hz, 3H). ^{13}C NMR (126 MHz, $CDCl_3$) δ 170.6, 170.4, 170.2, 169.6, 167.3, 137.7, 134.3, 127.8, 120.2, 86.8, 74.5, 72.1, 67.3 (2C), 61.7, 50.7, 44.6, 26.5, 22.8, 21.2, 21.0, 20.9, 20.8, 20.7. MS (ESI^+) m/z calculated for $[C_{26}H_{35}BrNO_{10}S]^+$ 632.12 $[M+H]^+$, found 632.00.

(2*R*,3*S*,4*S*,5*R*,6*S*)-2-(Acetoxymethyl)-6-((4-(2-(acetylthio)-3-phenylpropanamido)phenyl)thio)tetrahydro-2*H*-pyran-3,4,5-triyl triacetate (9)

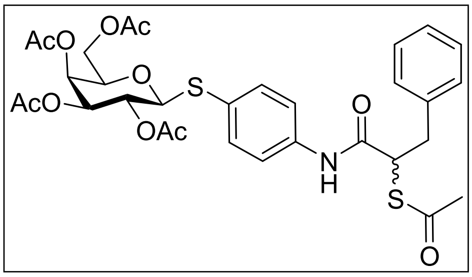

Compound **9** was synthesized according to the general procedure B, using compound **7** (147.7 mg, 0.22 mmol), potassium thioacetate (51 mg, 0.44 mmol) and acetone (5 mL). The crude product was purified using column chromatography (Hex/EtOAc = 1:1) to give compound **9** as a pale-yellow solid (106.2 mg, 72%). ^{1}H NMR (500 MHz, $CDCl_3$) δ 8.05 (bd, *J* = 4.4 Hz, 1H), 7.50 – 7.40 (m, 4H), 7.34 – 7.20 (m, 5H), 5.39 (bd, *J* = 3.4 Hz, 1H), 5.18 (td, *J* = 10.0, 2.8 Hz, 1H), 5.02 (dd, *J* = 9.9, 3.2 Hz, 1H), 4.60 (d, *J* = 9.9 Hz, 1H), 4.27 (dd, *J* = 8.3, 7.2 Hz, 1H),

4.20 – 4.05 (m, 2H), 3.89 (t, *J* = 6.6 Hz, 1H), 3.43 (dd, *J* = 14.1, 8.5 Hz, 1H), 2.99 (ddd, *J* = 14.2, 7.0, 1.8 Hz, 1H), 2.38 (s, 3H), 2.10 (s, 3H), 2.10 (s, 3H), 2.04 (s, 3H), 1.97 (s, 3H). ^{13}C NMR (126 MHz, $CDCl_3$) δ 197.70, 197.66 (two signals at 197.7 due to diastereomeric mixture), 170.6, 170.4, 170.2, 169.6, 168.6, 138.2, 137.6, 134.41, 134.37 (two signals at 134.4 due to diastereomeric mixture), 129.4, 128.8, 127.22, 127.17, 127.13 (two signals at 127.17 and 127.13 due to diastereomeric mixture), 120.17, 120.13 (two signals at 120.17 and 120.13 due to diastereomeric mixture), 87.0, 86.9 (two signals at 87.0 and 86.9 due to diastereomeric mixture), 74.6, 72.1, 67.4, 67.3, 61.7, 48.6, 48.5 (two signals at 48.6 and 48.5 due to diastereomeric mixture), 35.7, 30.6, 21.0, 20.86, 20.84 (two signals at 20.86 and 20.84 due to diastereomeric mixture), 20.81, 20.79 (two signals at 20.8 due to diastereomeric mixture), 20.73. MS (ESI$^+$) m/z calculated for $[C_{31}H_{36}NO_{11}S_2]^+$ 662.17 [M+H]$^+$, found 662.03.

(2*R*,3*S*,4*S*,5*R*,6*S*)-2-(Acetoxymethyl)-6-((4-(2-(acetylthio)-4-methylpentanamido)phenyl)thio)tetrahydro-2*H*-pyran-3,4,5-triyl triacetate (10)

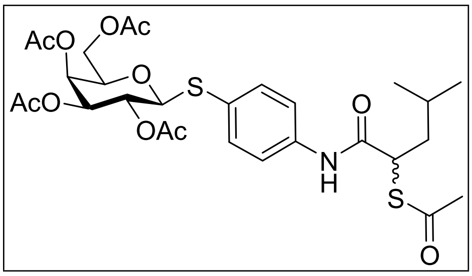

Compound **10** was synthesized according to the general procedure B, using compound **8** (152 mg, 0.24 mmol) and potassium thioacetate (55 mg, 0.48 mmol) in acetone (5 mL). The crude residue was purified using column chromatography (Hex/EtOAc = 8:2 to 6:4) to give compound **10** as a white solid (102 mg, 68%). ^{1}H NMR (500 MHz, $CDCl_3$) δ 8.23 (s, 1H), 7.53 – 7.44 (m, 4H), 5.40 (dd, *J* = 3.3, 0.8 Hz, 1H), 5.19 (t, *J* = 10.0 Hz, 1H), 5.02 (dd, *J* = 10.0, 3.3 Hz, 1H), 4.60 (d, *J* = 10.0 Hz, 1H), 4.20 – 4.14 (m, 1H), 4.12 – 4.06 (m, 2H), 3.91 – 3.86 (m, 1H), 2.41 (s, 3H), 2.12 (s, 3H), 2.10 (s, 3H), 2.05 (s, 3H), 2.01 – 1.94 (m, 1H), 1.98 (s, 3H), 1.73 (sep, *J* = 6.7 Hz, 1H), 1.59 – 1.54 (m, 1H), 0.96 (d, *J* = 6.6 Hz, 3H), 0.92 (d, *J* = 6.6 Hz, 3H). ^{13}C NMR (126 MHz, $CDCl_3$) δ 198.5, 170.6, 170.4, 170.2, 169.6, 169.3, 138.4, 134.4, 126.9, 120.0, 87.0, 74.5, 72.1, 67.3 (2C), 61.7, 45.2, 37.8, 30.5, 26.0, 22.5, 22.4, 21.0, 20.9, 20.84, 20.76. MS (ESI$^+$) m/z calculated for $[C_{28}H_{38}NO_{11}S_2]^+$ 628.19 [M+H]$^+$, found 628.06.

2-Mercapto-3-phenyl-*N*-(4-(((2*S*,3*R*,4*S*,5*R*,6*R*)-3,4,5-trihydroxy-6-(hydroxymethyl)tetrahydro-2*H*-pyran-2-yl)thio)phenyl)propanamide (11)

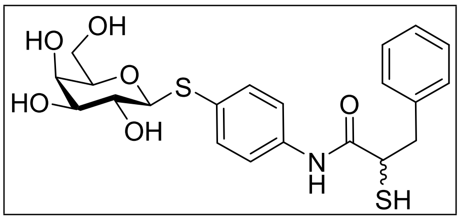

Compound **11** was synthesized according to the general procedure C, using compound **9** (102.7 mg, 0.16 mmol), sodium methoxide (175 µL, 5.33 M, 0.93 mmol) and MeOH (5 mL). The crude product was purified using preparative HPLC (CH_3CN (FA 0.05%)/H_2O (FA 0.05%) = 5:95 to 95:5), to give the compound **11** as a white solid (29.9 mg, 43%). ^1H NMR (500 MHz, DMSO-d_6) δ 10.05 (s, 1H), 7.51 – 7.44 (m, 2H), 7.39 (d, J = 8.6 Hz, 2H), 7.30 – 7.22 (m, 4H), 7.21 – 7.16 (m, 1H), 5.07 (d, J = 5.8 Hz, 1H), 4.83 (d, J = 5.6 Hz, 1H), 4.60 (t, J = 5.6 Hz, 1H), 4.45 (d, J = 9.3 Hz, 1H), 4.42 (d, J = 4.4 Hz, 1H), 3.76 – 3.70 (m, 1H), 3.68 (t, J = 3.7 Hz, 1H), 3.53 – 3.44 (m, 2H), 3.43 – 3.39 (m, 1H), 3.39 – 3.29 (m, 2H), 3.24 (dd, J = 13.6, 8.8 Hz, 1H), 3.12 (s, 1H), 2.93 (dd, J = 13.6, 6.4 Hz, 1H). ^{13}C NMR (126 MHz, DMSO-d_6) δ 170.4, 138.6, 137.6, 131.15, 131.13 (two signals at 131.15 and 131.13 due to diastereomeric mixture), 129.0, 128.9, 128.3, 126.5, 119.5, 88.27, 88.23 (two signals at 88.27 and 88.23 due to diastereomeric mixture), 79.2, 74.7, 69.2, 68.4, 60.6, 43.2, 41.2. HRMS (ESI$^-$) m/z calculated for $[C_{21}H_{24}NO_6S_2]^-$ 450.1051 $[M–H]^-$, found 450.1045.

2-Mercapto-4-methyl-*N*-(4-(((2*S*,3*R*,4*S*,5*R*,6*R*)-3,4,5-trihydroxy-6-(hydroxymethyl)tetrahydro-2*H*-pyran-2-yl)thio)phenyl)pentanamide (12).

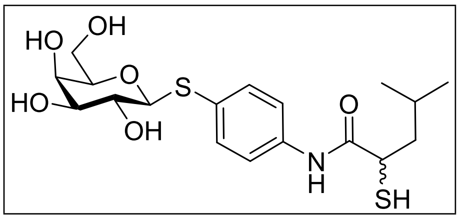

Compound **12** was synthesized according to the general procedure C, using compound **10** (97.4 mg, 0.16 mmol), sodium methoxide (175 µL, 5.33 M, 0.93 mmol) and MeOH (6 mL). The crude product was purified using preparative HPLC (CH_3CN (FA 0.05%)/H_2O (FA 0.05%) = 5:95 to 95:5), to give compound **12** as a white solid (38.9 mg, 60%). ^1H NMR (500 MHz, DMSO-d_6) δ 10.13 (s, 1H), 7.53 (d, J = 8.7 Hz, 2H), 7.42 (d, J = 8.7 Hz, 2H), 5.11 (d, J = 5.8, 1H), 4.88 (d, J = 5.5 Hz, 1H), 4.63 (t, J = 5.6 Hz, 1H), 4.48 – 4.44 (m, 2H), 3.68 (t, J = 3.7 Hz, 1H), 3.55 – 3.37 (m, 5H), 3.34 – 3.29 (m, 1H), 3.01 (s, 1H), 1.79 (ddd, J = 13.3, 8.5, 6.4 Hz, 1H), 1.62 (sep, J = 6.7 Hz, 1H), 1.50 (dt, J = 13.7, 7.0 Hz, 1H), 0.91 (d, J = 6.6 Hz, 3H), 0.86 (d, J = 6.6 Hz, 3H). ^{13}C NMR (126 MHz, DMSO-d_6) δ 171.2, 137.8, 131.2, 128.8, 119.5, 88.3, 79.2, 74.7, 69.2, 68.4, 60.6, 44.3, 40.1 (behind DMSO, based on HSQC), 25.8, 22.3, 22.1. HRMS (ESI$^+$) m/z calculated for $[C_{18}H_{28}NO_6S_2]^+$ 418.1353 $[M+H]^+$, found 418.1339.

(2*R*,3*S*,4*S*,5*R*,6*S*)-2-(Acetoxymethyl)-6-((4-((*S*)-2-bromo-3-phenylpropanamido)phenyl)thio)tetrahydro-2*H*-pyran-3,4,5-triyl triacetate (13)

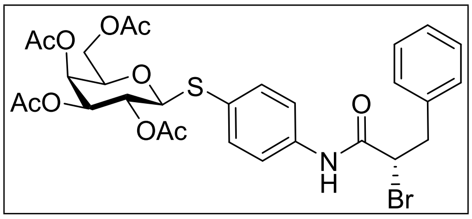

Compound **13** was synthesized according to the general procedure A, using (*S*)-2-bromo-3-phenylpropanoic acid (120.7 mg, 0.53 mmol), compound **6** (200 mg, 0.44 mmol), EDC·HCl (101.3 mg, 0.53 mmol) and DCM (15 mL). The obtained crude product was purified using column chromatography (Hex/EtOAc = 6:4 to 1:1), to give compound **13** as a white solid (246.9 mg, 84%). ^{1}H NMR (500 MHz, $CDCl_3$) δ 7.85 (s, 1H), 7.52 – 7.48 (m, 2H), 7.46 – 7.42 (m, 2H), 7.34 – 7.24 (m, 5H), 5.41 (d, *J* = 2.6 Hz, 1H), 5.20 (t, *J* = 10.0 Hz, 1H), 5.04 (dd, *J* = 9.9, 3.4 Hz, 1H), 4.64 (d, *J* = 9.9 Hz, 1H), 4.61 (dd, *J* = 7.5, 6.0 Hz, 1H), 4.21 – 4.15 (m, 1H), 4.13 – 4.08 (m, 1H), 3.91 (t, *J* = 6.6 Hz, 1H), 3.61 (dd, *J* = 14.3, 5.8 Hz, 1H), 3.35 (dd, *J* = 14.3, 7.5 Hz, 1H), 2.11 (s, 3 H), 2.10 (s, 3 H), 2.05 (s, 3 H), 1.97 (s, 3 H). ^{13}C NMR (126 MHz, $CDCl_3$) δ 170.5, 170.3, 170.2, 169.6, 166.2, 137.4, 136.7, 134.2, 129.6, 128.7, 128.1, 127.6, 120.4, 86.8, 74.6, 72.1, 67.33, 67.31, 61.7, 51.8, 41.7, 21.0, 20.9, 20.8, 20.7. MS (ESI$^+$) m/z calculated for $[C_{29}H_{33}BrNO_{10}S]^+$ 666.10 $[M+H]^+$, found 666.12.

(2*R*,3*S*,4*S*,5*R*,6*S*)-2-(Acetoxymethyl)-6-((4-((*S*)-2-bromo-4-methylpentanamido)phenyl)thio)tetrahydro-2*H*-pyran-3,4,5-triyl triacetate (14)

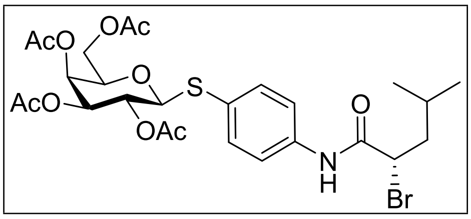

Compound **14** was synthesized according to the general procedure A, using (*S*)-2-bromo-4-methylpentanoic acid (103 mg, 0.53 mmol), EDC·HCl (101 mg, 0.53 mmol), aniline **6** (200 mg, 0.44 mmol) and DCM (20 mL). The crude product obtained was purified using column chromatography (Hex/EtOAc = 1:1), to give compound **14** as a white solid (232 mg, 84%). ^{1}H NMR (500 MHz, $CDCl_3$) δ 8.04 (s, 1H), 7.51 (s, 4H), 5.41 (d, *J* = 2.9 Hz, 1H), 5.20 (t, *J* = 10.0 Hz, 1H), 5.03 (dd, *J* = 9.9, 3.4 Hz, 1H), 4.63 (d, *J* = 9.9 Hz, 1H), 4.43 (dd, *J* = 9.5, 5.5 Hz, 1H), 4.21 – 4.15 (m, 1H), 4.13 – 4.08 (m, 1H), 3.91 (t, *J* = 6.6 Hz, 1H), 2.12 (s, 3H), 2.10 (s, 3H), 2.05 (s, 3H), 2.04 – 1.95 (m, 2H), 1.97 (s, 3H), 1.94 – 1.85 (m, 1H), 1.00 (d, *J* = 6.7 Hz, 3H), 0.94 (d, *J* = 6.6 Hz, 3H). ^{13}C NMR (126 MHz, $CDCl_3$) δ 170.6, 170.4, 170.2, 169.6, 167.2, 137.7, 134.3, 127.8, 120.2, 86.8, 74.5, 72.1, 67.27, 67.26, 61.7, 50.7, 44.6, 26.5, 22.8, 21.2, 21.0, 20.9, 20.83, 20.75. MS (ESI$^-$) m/z calculated for $[C_{26}H_{33}BrNO_{10}S]^-$ 630.10 $[M–H]^-$, found 630.28.

(2*R*,3*S*,4*S*,5*R*,6*S*)-2-(Acetoxymethyl)-6-((4-((*R*)-2-(acetylthio)-3-phenylpropanamido)phenyl)thio)tetrahydro-2*H*-pyran-3,4,5-triyl triacetate (15)

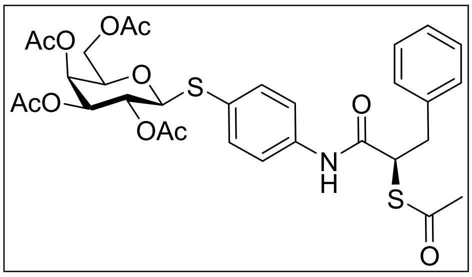

Compound **15** was synthesized according to the general procedure B, using compound **13** (239.3 mg, 0.36 mmol), potassium thioacetate (82 mg, 0.72 mmol) and acetone (7 mL). The crude product was purified using column chromatography (Hex/EtOAc = 1:1) to give compound **15** as a white solid (198.4 mg, 84%). ^{1}H NMR (500 MHz, $CDCl_3$) δ 8.06 (s, 1H), 7.48 – 7.40 (m, 4H), 7.32 – 7.20 (m, 5H), 5.40 (d, *J* = 2.9 Hz, 1H), 5.19 (t, *J* = 10.0 Hz, 1H), 5.02 (dd, *J* = 9.9, 3.4 Hz, 1H), 4.60 (d, *J* = 9.9 Hz, 1H), 4.27 (dd, *J* = 8.5, 7.1 Hz, 1H), 4.19 – 4.14 (m, 1H), 4.13 – 4.07 (m, 1H), 3.89 (t, *J* = 6.7 Hz, 1H), 3.43 (dd, *J* = 14.2, 8.5 Hz, 1H), 2.98 (dd, *J* = 14.1, 7.1 Hz, 1H), 2.38 (s, 3H), 2.11 (s, 3H), 2.10 (s, 3H), 2.04 (s, 3H), 1.97 (s, 3H). ^{13}C NMR (126 MHz, $CDCl_3$) δ 197.7, 170.5, 170.4, 170.2, 169.6, 168.5, 138.2, 137.6, 134.4, 129.4, 128.8, 127.2, 127.1, 120.1, 86.9, 74.6, 72.1, 67.4, 67.3, 61.7, 48.5, 35.7, 30.6, 21.0, 20.84, 20.80, 20.7. MS (ESI$^+$) m/z calculated for $[C_{31}H_{36}NO_{11}S_2]^+$ 662.17 [M+H]$^+$, found 662.23.

(2*R*,3*S*,4*S*,5*R*,6*S*)-2-(Acetoxymethyl)-6-((4-((*R*)-2-(acetylthio)-4-methylpentanamido)phenyl)thio)tetrahydro-2*H*-pyran-3,4,5-triyl triacetate (16)

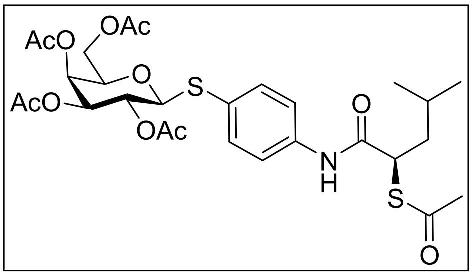

Compound **16** was synthesized according to the general procedure B, using compound **14** (225 mg, 0.36 mmol) and potassium thioacetate (81.2 mg, 0.71 mmol) in acetone (7 mL). The crude residue was purified using column chromatography (Hex/EtOAc = 8/2 to 6/4) to give compound **16** as a white solid (151 mg, 68%). ^{1}H NMR (500 MHz, $CDCl_3$) δ 8.23 (s, 1H), 7.52 – 7.45 (m, 4H), 5.40 (d, *J* = 2.7 Hz, 1H), 5.19 (t, *J* = 9.9 Hz, 1H), 5.02 (dd, *J* = 9.9, 3.4 Hz, 1H), 4.60 (d, *J* = 9.9 Hz, 1H), 4.20 – 4.14 (m, 1H), 4.13 – 4.06 (m, 2H), 3.91 – 3.87 (m, 1H), 2.41 (s, 3H), 2.12 (s, 3H), 2.10 (s, 3H), 2.05 (s, 3H), 2.00 – 1.95 (m, 1H), 1.98 (s, 3H), 1.74 (sep, *J* = 6.7 Hz, 1H), 1.60 – 1.54 (m, 1H), 0.96 (d, *J* = 6.6 Hz, 3H), 0.92 (d, *J* = 6.6 Hz, 3H). ^{13}C NMR (126 MHz, $CDCl_3$) δ 198.5, 170.6, 170.4, 170.2, 169.6, 169.3, 138.4, 134.4, 126.9, 120.0, 87.0, 74.5, 72.1, 67.3 (2C), 61.7, 45.2, 37.8, 30.5, 26.0, 22.5, 22.4, 21.0, 20.9, 20.84, 20.76. MS (ESI$^+$) m/z calculated for $[C_{28}H_{38}NO_{11}S_2]^+$ 628.19 [M+H]$^+$, found 628.13.

(*R*)-2-Mercapto-3-phenyl-*N*-(4-(((2*S*,3*R*,4*S*,5*R*,6*R*)-3,4,5-trihydroxy-6-(hydroxymethyl)tetrahydro-2*H*-pyran-2-yl)thio)phenyl)propanamide (17)

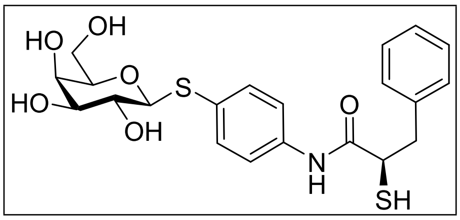

Compound **17** was synthesized according to the general procedure C, using compound **15** (184.3 mg, 0.28 mmol), sodium methoxide (210 µL, 5.33 M in MeOH, 1.11 mmol) and MeOH (6 mL). The crude product was purified using preparative HPLC (CH_3CN (FA 0.05%)/H_2O (FA 0.05%) = 5/95 to 95/5), to give the compound **17** as a white solid (47 mg, 37%). ^{1}H NMR (500 MHz, DMSO-d_6) δ 10.05 (s, 1H), 7.46 (d, *J* = 8.7 Hz, 2H), 7.39 (d, *J* = 8.7 Hz, 2H), 7.30 – 7.21 (m, 4H), 7.21 – 7.16 (m, 1H), 5.07 (d, *J* = 5.8 Hz, 1H), 4.83 (d, *J* = 5.6 Hz, 1H), 4.61 (t, *J* = 5.6 Hz, 1H), 4.45 (d, *J* = 9.3 Hz, 1H), 4.42 (d, *J* = 4.4 Hz, 1H), 3.75 – 3.71 (m, 1H), 3.68 (t, *J* = 3.7 Hz, 1H), 3.53 – 3.44 (m, 2H), 3.43 – 3.39 (m, 1H), 3.39 – 3.29 (m, 2H), 3.23 (dd, *J* = 13.6, 8.8 Hz, 1H), 3.12 (s, 1H), 2.93 (dd, *J* = 13.6, 6.4 Hz, 1H). ^{13}C NMR (126 MHz, DMSO-d_6) δ 170.5, 138.6, 137.6, 131.2, 129.0, 128.9, 128.3, 126.5, 119.5, 88.27, 79.2, 74.7, 69.2, 68.4, 60.6, 43.2, 41.2. HRMS (ESI$^-$) m/z calculated for $[C_{21}H_{24}NO_6S_2]^-$ 450.1051 [M–H]$^-$, found 450.1051.

(*R*)-2-Mercapto-4-methyl-*N*-(4-(((2*S*,3*R*,4*S*,5*R*,6*R*)-3,4,5-trihydroxy-6-(hydroxymethyl)tetrahydro-2*H*-pyran-2-yl)thio)phenyl)pentanamide (18).

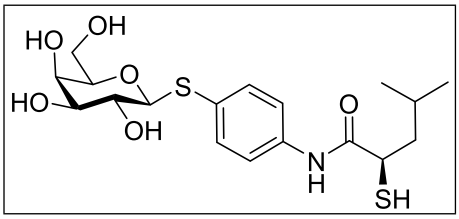

Compound **18** was synthesized according to the general procedure C, using compound **16** (136.8 mg, 0.22 mmol), sodium methoxide (245 µL, 5.33 M, 1.31 mmol) and MeOH (6 mL). The crude product was purified using preparative HPLC (CH_3CN (FA 0.05%)/H_2O (FA 0.05%) = 5:95 to 95:5), to give compound **18** as a white solid (44.3 mg, 49%). ^{1}H NMR (500 MHz, DMSO-d_6) δ 10.14 (s, 1H), 7.53 (d, *J* = 8.7 Hz, 2H), 7.42 (d, *J* = 8.7 Hz, 2H), 5.12 (d, *J* = 5.8 Hz, 1H), 4.88 (d, *J* = 5.5 Hz, 1H), 4.65 (t, *J* = 5.6 Hz, 1H), 4.48 – 4.44 (m, 2H), 3.68 (t, *J* = 3.7 Hz, 1H), 3.54 – 3.44 (m, 3H), 3.43 – 3.35 (m, 2H), 3.35 – 3.29 (m, 1H), 3.00 (s, 1H), 1.79 (ddd, *J* = 13.6, 8.4, 6.5 Hz, 1H), 1.61 (sep, *J* = 6.7 Hz, 1H), 1.50 (dt, *J* = 13.7, 7.0 Hz, 1H), 0.90 (d, *J* = 6.6 Hz, 3H), 0.85 (d, *J* = 6.6 Hz, 3H). ^{13}C NMR (126 MHz, DMSO-d_6) δ 171.2, 137.8, 131.3, 128.8, 119.5, 88.3, 79.2, 74.7, 69.2, 68.4, 60.6, 44.3, 40.1 (behind DMSO, based on HSQC), 25.8, 22.3, 22.2. HRMS (ESI$^-$) m/z calculated for $[C_{18}H_{26}NO_6S_2]^-$ 416.1207 [M–H]$^-$, found 416.1210.

(*R*)-2-Bromo-4-methylpentanoic acid (19′)

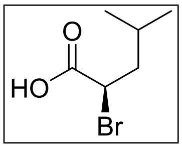

(*R*)-2-Bromo-4-methylpentanoic acid **19′** was synthesized according to the protocol reported in literature.[15] **19′** was obtained as colorless oil in quantitative yield and used in the next step without further purification.

(2*R*,3*S*,4*S*,5*R*,6*S*)-2-(Acetoxymethyl)-6-((4-((*R*)-2-bromo-3-phenylpropanamido)phenyl)thio)tetrahydro-2*H*-pyran-3,4,5-triyl triacetate (20)

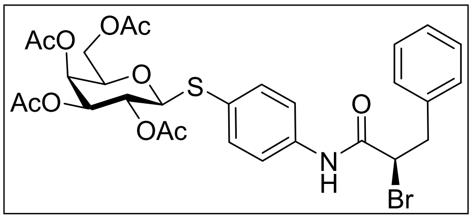

Compound **20** was synthesized according to the general procedure A, using (*R*)-2-bromo-3-phenylpropanoic acid **19** (120 mg, 0.52 mmol), compound **6** (197.4 mg, 0.43 mmol), EDC·HCl (100.1 mg, 0.52 mmol) and DCM (15 mL). The obtained crude product was purified using column chromatography (Hex/EtOAc = 6:4 to 1:1), to give compound **20** as a white solid (261.3 mg, 91%). ^{1}H NMR (500 MHz, $CDCl_3$) δ 7.85 (s, 1H), 7.52 – 7.48 (m, 2H), 7.46 – 7.42 (m, 2H), 7.34 – 7.24 (m, 5H), 5.41 (d, *J* = 2.9 Hz, 1H), 5.20 (t, *J* = 10.0 Hz, 1H), 5.04 (dd, *J* = 9.9, 3.4 Hz, 1H), 4.64 (d, *J* = 9.9 Hz, 1H), 4.60 (dd, *J* = 7.4, 6.0 Hz, 1H), 4.20 – 4.15 (m, 1H), 4.13 – 4.08 (m, 1H), 3.91 (t, *J* = 6.6 Hz, 1H), 3.61 (dd, *J* = 14.4, 5.9 Hz, 1H), 3.34 (dd, *J* = 14.3, 7.5 Hz, 1H), 2.11 (s, 3 H), 2.10 (s, 3 H), 2.05 (s, 3 H), 1.97 (s, 3 H). ^{13}C NMR (126 MHz, $CDCl_3$) δ 170.5, 170.3, 170.2, 169.6, 166.2, 137.4, 136.7, 134.2, 129.6, 128.7, 128.1, 127.6, 120.4, 86.8, 74.6, 72.1, 67.34, 67.31, 61.7, 51.8, 41.7, 21.0, 20.9, 20.8, 20.7. MS (ESI$^+$) m/z calculated for $[C_{29}H_{33}BrNO_{10}S]^+$ 666.10 $[M+H]^+$, found 666.11.

(2*R*,3*S*,4*S*,5*R*,6*S*)-2-(Acetoxymethyl)-6-((4-((*R*)-2-bromo-4-methylpentanamido)phenyl)thio)tetrahydro-2*H*-pyran-3,4,5-triyl triacetate (21)

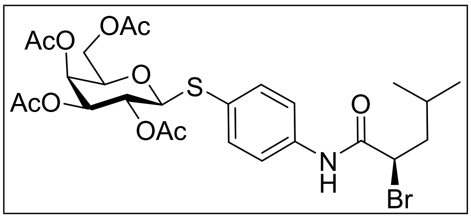

Compound **21** was synthesized according to the general procedure A, using (*R*)-2-bromo-4-methylpentanoic acid **19** (160.6 mg, 0.82 mmol), EDC·HCl (156.7 mg, 0.82 mmol), aniline **6** (250 mg, 0.55 mmol) and DCM (15 mL). The obtained crude product was purified using column chromatography (Hex/EtOAc = 1:1), to give compound **21** as a pale-yellow solid (240 mg, 69%). ^{1}H NMR (500 MHz, $CDCl_3$) δ 8.03 (s, 1H), 7.51 (s, 4H), 5.41 (d, *J* = 3.2 Hz, 1H), 5.20 (t, *J* = 9.9 Hz, 1H), 5.04 (dd, *J* = 10.0, 3.3 Hz, 1H), 4.64 (d, *J* = 9.9 Hz, 1H), 4.43 (dd, *J* = 9.4, 5.4 Hz, 1H), 4.20 – 4.15 (m, 1H), 4.14 – 4.08 (m, 1H), 3.91 (t, *J* = 6.6 Hz, 1H), 2.11 (s, 3H), 2.10 (s, 3H), 2.05 (s, 3H), 2.04 – 1.96 (m, 2H), 2.00 (s, 3H), 1.94 – 1.85 (m, 1H), 1.00 (d, *J* = 6.6 Hz, 3H), 0.95 (d, *J* = 6.6 Hz, 3H). ^{13}C

NMR (126 MHz, $CDCl_3$) δ 170.5, 170.3, 170.2, 169.6, 167.2, 137.7, 134.4, 127.8, 120.2, 86.8, 74.6, 72.1, 67.33, 67.32, 61.7, 50.6, 44.6, 26.6, 22.8, 21.2, 21.0, 20.9, 20.8, 20.7. MS (ESI^+) m/z calculated for $[C_{26}H_{35}BrNO_{10}S]^+$ 632.12 $[M+H]^+$, found 632.12.

(2*R*,3*S*,4*S*,5*R*,6*S*)-2-(Acetoxymethyl)-6-((4-((*S*)-2-(acetylthio)-3-phenylpropanamido)phenyl)thio)tetrahydro-2*H*-pyran-3,4,5-triyl triacetate (22)

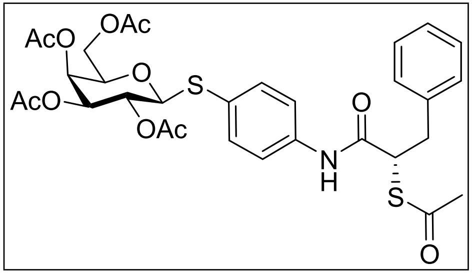

Compound **22** was synthesized according to the general procedure B, using compound **20** (246.5 mg, 0.37 mmol), potassium thioacetate (84.5 mg, 0.74 mmol) and acetone (6 mL). The crude product was purified using column chromatography (Hex/EtOAc = 1:1) to give compound **15** as a white solid (219.2 mg, 89%). ^1H NMR (500 MHz, $CDCl_3$) δ 8.03 (s, 1H), 7.48 – 7.40 (m, 4H), 7.32 – 7.22 (m, 5H), 5.39 (dd, *J* = 3.4, 0.8 Hz, 1H), 5.18 (t, *J* = 9.9 Hz, 1H), 5.02 (dd, *J* = 10.1, 3.4 Hz, 1H), 4.60 (d, *J* = 9.9 Hz, 1H), 4.27 (dd, *J* = 8.4, 7.2 Hz, 1H), 4.19 – 4.13 (m, 1H), 4.13 – 4.08 (m, 1H), 3.91 – 3.86 (m, 1H), 3.43 (dd, *J* = 14.2, 8.5 Hz, 1H), 2.99 (dd, *J* = 14.0, 7.0 Hz, 1H), 2.38 (s, 3H), 2.09 (s, 6H), 2.04 (s, 3H), 1.97 (s, 3H). ^{13}C NMR (126 MHz, $CDCl_3$) δ 197.6, 170.5, 170.3, 170.2, 169.6, 168.5, 138.2, 137.6, 134.4, 129.4, 128.8, 127.22, 127.17, 120.2, 87.0, 74.6, 72.1, 67.4, 67.3, 61.7, 48.5, 35.7, 30.6, 21.0, 20.9, 20.7, 20.7. MS (ESI^+) m/z calculated for $[C_{31}H_{36}NO_{11}S_2]^+$ 662.17 $[M+H]^+$, found 662.24.

(2*R*,3*S*,4*S*,5*R*,6*S*)-2-(Acetoxymethyl)-6-((4-((*S*)-2-(acetylthio)-4-methylpentanamido)phenyl)thio)tetrahydro-2*H*-pyran-3,4,5-triyl triacetate (23)

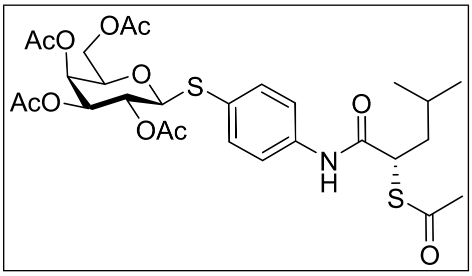

Compound **23** was synthesized according to the general procedure B, using compound **21** (227 mg, 0.36 mmol) and potassium thioacetate (81.8 mg, 0.72 mmol) in acetone (7 mL). The crude residue was purified using column chromatography (Hex/EtOAc = 8:2 to 6:4) to give compound **23** as a pale-yellow solid (188 mg, 84%). ^1H NMR (500 MHz, $CDCl_3$) δ 8.21 (s, 1H), 7.52 – 7.44 (m, 4H), 5.41 – 5.38 (m, 1H), 5.19 (t, *J* = 10.0 Hz, 1H), 5.03 (dd, *J* = 9.9, 3.4 Hz, 1H), 4.61 (d, *J* = 9.9 Hz, 1H), 4.20 – 4.14 (m, 1H), 4.13 – 4.06 (m, 2H), 3.91 – 3.87 (m, 1H), 2.41 (s, 3H), 2.11 (s, 3H), 2.10 (s, 3H), 2.05 (s, 3H), 2.02 – 1.94 (m, 1H), 1.97 (s, 3H), 1.74 (sep, *J* = 6.7 Hz, 1H), 1.62 – 1.54 (m, 1H), 0.97 (d, *J* = 6.6 Hz, 3H), 0.93 (d, *J* = 6.6 Hz, 3H). ^{13}C NMR (126 MHz, $CDCl_3$) δ 198.4, 170.6, 170.4, 170.2, 169.6, 169.3, 138.5, 134.5,

127.0, 120.0, 87.1, 74.6, 72.1, 67.4, 67.3, 61.7, 45.3, 37.9, 30.5, 26.0, 22.5, 22.4, 21.0, 20.9, 20.8, 20.7. MS (ESI$^+$) m/z calculated for $[C_{28}H_{38}NO_{11}S_2]^+$ 628.19 [M+H]$^+$, found 628.22.

(*S*)-2-Mercapto-3-phenyl-*N*-(4-(((2*S*,3*R*,4*S*,5*R*,6*R*)-3,4,5-trihydroxy-6-(hydroxymethyl)tetrahydro-2*H*-pyran-2-yl)thio)phenyl)propanamide (24)

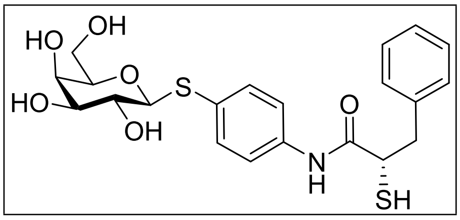

Compound **24** was synthesized according to the general procedure C, using compound **22** (201.8 mg, 0.30 mmol), sodium methoxide (230 µL, 5.33 M, 1.22 mmol) and MeOH (6 mL). After the workup with Amberlite, the mixture was evaporated, the obtained solid washed with MeOH and dried under high vacuum to afford the compound 24 as white solid (81 mg, 60%). ^{1}H NMR (500 MHz, DMSO-d_6) δ 10.04 (s, 1H), 7.47 (d, *J* = 8.7 Hz, 2H), 7.39 (d, *J* = 8.7 Hz, 2H), 7.30 – 7.22 (m, 4H), 7.21 – 7.17 (m, 1H), 5.07 (d, *J* = 5.8 Hz, 1H), 4.83 (d, *J* = 5.6 Hz, 1H), 4.60 (t, *J* = 5.6 Hz, 1H), 4.45 (d, *J* = 9.4 Hz, 1H), 4.42 (d, *J* = 4.4 Hz, 1H), 3.76 – 3.70 (m, 1H), 3.68 (t, *J* = 3.7 Hz, 1H), 3.53 – 3.44 (m, 2H), 3.43 – 3.39 (m, 1H), 3.39 – 3.34 (m, 1H), 3.32 – 3.29 (m, 1H), 3.24 (dd, *J* = 13.6, 8.8 Hz, 1H), 3.12 (s, 1H), 2.93 (dd, *J* = 13.6, 6.4 Hz, 1H). ^{13}C NMR (126 MHz, DMSO-d_6) δ 170.4, 138.6, 137.6, 131.1, 129.0, 128.8, 128.2, 126.5, 119.5, 88.23, 79.2, 74.7, 69.2, 68.3, 60.6, 43.2, 41.2. HRMS (ESI$^-$) m/z calculated for $[C_{21}H_{24}NO_6S_2]^-$ 450.1051 [M–H]$^-$, found 450.1044.

(*S*)-2-Mercapto-4-methyl-*N*-(4-(((2*S*,3*R*,4*S*,5*R*,6*R*)-3,4,5-trihydroxy-6-(hydroxymethyl)tetrahydro-2*H*-pyran-2-yl)thio)phenyl)pentanamide (25).

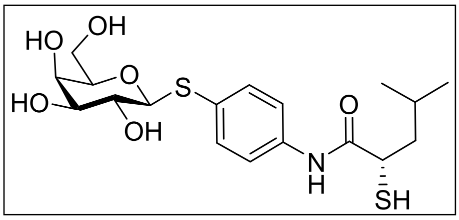

Compound **25** was synthesized according to the general procedure C, using compound **23** (183.8 mg, 0.29 mmol), sodium methoxide (220 µL, 5.33 M, 1.17 mmol) and MeOH (6 mL). The crude product was purified using preparative HPLC (CH_3CN (FA 0.05%)/H_2O (FA 0.05%) = 5:95 to 95:5), to give compound **25** as a white solid (82.3 mg, 68%). ^{1}H NMR (500 MHz, DMSO-d_6) δ 10.12 (s, 1H), 7.53 (d, *J* = 8.7 Hz, 2H), 7.42 (d, *J* = 8.7 Hz, 2H), 5.08 (d, *J* = 5.7, 1H), 4.84 (d, *J* = 5.5 Hz, 1H), 4.61 (t, *J* = 5.5 Hz, 1H), 4.48 – 4.42 (m, 2H), 3.69 (t, *J* = 3.6 Hz, 1H), 3.55 – 3.45 (m, 3H), 3.44 – 3.40 (m, 1H), 3.39 – 3.30 (m, 2H), 2.98 (s, 1H), 1.83 – 1.75 (m, 1H), 1.62 (sep, *J* = 6.7 Hz, 1H), 1.50 (dt, *J* = 13.7, 7.0 Hz, 1H), 0.91 (d, *J* = 6.6 Hz, 3H), 0.86 (d, *J* = 6.6 Hz, 3H). ^{13}C NMR (126 MHz, DMSO-d_6) δ 171.2, 137.8, 131.3, 128.8, 119.5,

88.3, 79.2, 74.7, 69.2, 68.4, 60.6, 44.3, 40.1 (behind DMSO, based on HSQC), 25.8, 22.2, 22.1. HRMS (ESI$^-$) m/z calculated for $[C_{18}H_{26}NO_6S_2]^-$ 416.1207 [M–H]$^-$, found 416.1198.

2,2'-Disulfanediylbis(3-phenyl-*N*-(4-(((2*S*,3*R*,4*S*,5*R*,6*R*)-3,4,5-trihydroxy-6-(hydroxymethyl)tetrahydro-2*H*-pyran-2-yl)thio)phenyl)propanamide) (26a and 26b)

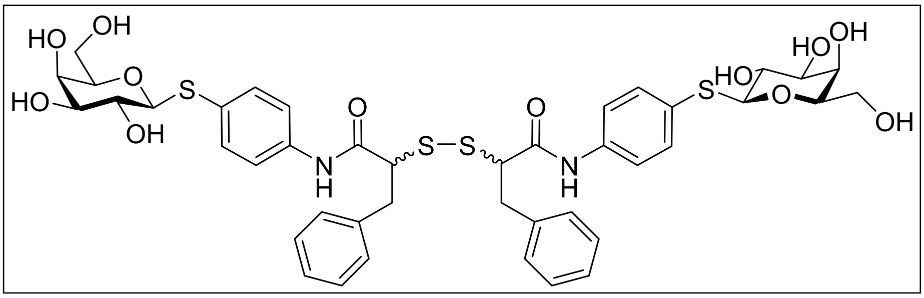

Compound **26** was synthesized according to the general procedure D, using thiol **11** (9.3 mg, 0.02 mmol), 3% (v/v) H_2O_2 (50 µL, 2.5 equiv., 0.05 mmol) and DMSO/H_2O 1:1 (500 µL). The product was purified by preparative HPLC (H_2O/MeCN + 0.1% formic acid, 30-60% MeCN) to result in two fractions **26a** (t_R 6.1 min) and **26b** (t_R 6.3 min). Both compounds were obtained as white solids (3.7 mg of **26a** and 4.1 mg of **26b**; 7.8 mg in total, 8.6 µmol, 86%). **26a:** ^{1}H NMR (500 MHz, DMSO-d_6) δ 10.16 (s, 1H), 10.10 (s, 1H), 7.52 – 7.36 (m, 8H), 7.31 – 7.10 (m, 10H), 5.09 (t, *J* = 5.6 Hz, 2H), 4.84 (dd, *J* = 5.7, 2.4 Hz, 2H), 4.60 (t, *J* = 5.5 Hz, 2H), 4.46 (d, *J* = 9.5 Hz, 2H), 4.45 – 4.43 (m, 2H), 4.00 – 3.94 (m, 2H), 3.69 (t, *J* = 3.7 Hz, 2H), 3.55 – 3.44 (m, 4H), 3.42 (d, *J* = 6.0 Hz, 2H), 3.40 – 3.34 (m, 2H), 3.31 – 3.29 (m, 2H), 3.28 – 3.18 (m, 2H), 3.12 (dd, *J* = 13.7, 5.5 Hz, 1H), 3.02 (dd, *J* = 13.7, 5.5 Hz, 1H). ^{13}C NMR (126 MHz, DMSO-d_6) δ 168.4, 168.3, 137.9, 137.8, 137.2, 137.1, 131.0, 130.9, 129.5, 129.2, 129.0, 128.9, 128.3, 126.6, 119.8, 119.7, 88.3, 79.2, 74.7, 69.2, 68.3, 60.5, 56.0, 55.2, 48.6, 37.1, 36.7. HRMS (ESI$^+$) m/z calculated for $[C_{42}H_{49}N_2O_{12}S_4]^+$ 901.2163 [M+H]$^+$, found 901.2157. **26b:** ^{1}H NMR (500 MHz, DMSO-d_6) δ 10.17 (s, 1H), 10.11 (s, 1H), 7.54 – 7.33 (m, 8H), 7.31 – 7.08 (m, 10H), 5.09 (t, *J* = 5.7 Hz, 2H), 4.84 (dd, *J* = 5.7, 2.5 Hz, 2H), 4.60 (t, *J* = 5.5 Hz, 2H), 4.46 (d, *J* = 9.5 Hz, 2H), 4.45 – 4.42 (m, 2H), 4.00 – 3.95 (m, 2H), 3.69 (t, *J* = 3.7 Hz, 2H), 3.55 – 3.44 (m, 4H), 3.42 (t, *J* = 6.4 Hz, 2H), 3.39 – 3.35 (m, 2H), 3.31 – 3.29 (m, 2H), 3.28 – 3.19 (m, 2H), 3.12 (dd, *J* = 13.7, 5.5 Hz, 1H), 3.02 (dd, *J* = 13.7, 5.5 Hz, 1H). ^{13}C NMR (126 MHz, DMSO-d_6) δ 168.4, 168.3, 137.9, 137.8, 137.2, 137.1, 131.0, 131.0, 129.5, 129.2, 129.0, 128.9, 128.3, 126.6, 119.8, 119.7, 88.3, 79.2, 74.7, 69.2, 68.3, 60.5, 56.0, 55.2, 48.6, 37.0, 36.7. HRMS (ESI$^+$) m/z calculated for $[C_{42}H_{49}N_2O_{12}S_4]^+$ 901.2163 [M+H]$^+$, found 901.2157.

2,2'-Disulfanediylbis(4-methyl-*N*-(4-(((2*S*,3*R*,4*S*,5*R*,6*R*)-3,4,5-trihydroxy-6-(hydroxymethyl)tetrahydro-2*H*-pyran-2-yl)thio)phenyl)pentanamide) (27).

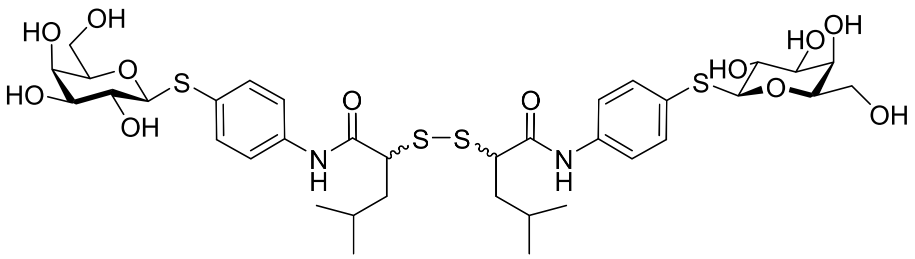

Compound **27** was synthesized according to the general procedure D, using thiol **12** (3.74 mg, 8.8 µmol), 3% (v/v) H_2O_2 (22 µL, 2.5 equiv., 22 µmol) and DMSO/H_2O 1:1 (200 µL). The product was purified by preparative HPLC (H_2O/MeCN + 0.1% formic acid, 15 - 55% MeCN) and **27** was obtained as a white solid (3 mg, 3.6 µmol, 82%). ^{1}H NMR (500 MHz, DMSO-d_6) δ 10.28 (s, 1H), 10.19 (s, 1H), 7.61 – 7.51 (m, 4H), 7.46 – 7.38 (m, 4H), 5.09 (dd, J = 5.9, 2.3 Hz, 2H), 4.84 (d, J = 5.6 Hz, 2H), 4.60 (t, J = 5.6 Hz, 2H), 4.46 (d, J = 9.5 Hz, 2H), 4.43 (d, J = 4.4 Hz, 2H), 3.78 – 3.71 (m, 2H), 3.69 (t, J = 3.9 Hz, 2H), 3.54 – 3.44 (m, 4H), 3.44 – 3.35 (m, 4H), 3.30 (s, 2H), 1.93 – 1.76 (m, 2H), 1.62 – 1.42 (m, 4H), 0.90 (d, J = 6.0 Hz, 3H), 0.87 (d, J = 6.0 Hz, 3H), 0.82 (d, J = 6.2 Hz, 3H), 0.80 (d, J = 6.2 Hz, 3H). ^{13}C NMR (126 MHz, DMSO-d_6) δ 169.3, 169.2, 137.5, 137.4, 131.1, 129.3, 129.0, 119.8, 119.7, 88.3, 79.2, 74.7, 69.2, 68.3, 60.5, 53.3, 48.6, 40.4, 40.2, 26.0, 25.9, 22.7, 22.6, 22.0, 21.8. HRMS (ESI$^+$) m/z calculated for $[C_{36}H_{53}N_2O_{12}S_4]^+$ 833.2476 [M+H]$^+$, found 833.2469.

Table S1. Inhibition of six human matrix metalloproteases (MMPs) in the presence of 100 µM of compounds **11** and **12***.

	Percent inhibition at 100 µM	
	**11**	**12**
MMP-1	10±5	n.i.
MMP-2	n.i.	19±3
MMP-3	n.i.	n.i.
MMP-7	n.i.	n.i.
MMP-8	n.i.	n.i.
MMP-14	n.i.	19±5

*Means and standard deviations from at least three independent experiments. n.i. = <10% inhibition

Table S2. Antibacterial activity of compounds **11** and **12** against *Pseudomonas aeruginosa* PA14.

	MIC (µM)	
	**11**	**12**
P. aeruginosa PA14	>100	>100

Table S3. Cytotoxicity of compounds **11** and **12** against HepG2, HEK293, and A549 cell lines*.

	IC_{50} (µM)	
	**11**	**12**
HepG2	>100	>100
HEK293	>100	>100
A549	>100	>100

*Means from at least three independent experiments

Table S4. Crystal structure of LasB in complex with **11**: Data collection and refinement statistics.

	**LasB_11**
**PDB ID**	**7Z68**

**Data collection**	
Space group	P 1 2_1 1
Cell dimension	
a, b, c (Å)	39.3, 92.5, 40.7
α, β, γ (°)	90.0, 114.2, 90
Wavelength (Å)	0.9763
Resolution	1.5 (1.53 – 1.50)
R_{sym} or R_{merge}	0.054 (0.318)
R_{pim}	0.031 (0.190)
CC (1/2)	0.999 (0.944)
I /σI	18.1 (4.7)
Completeness (%)	99.9 (99.3)
Redundancy	7.1 (6.3)
**Refinement**	
Resolution (Å)	37.16 – 1.5
No. reflection	42387
R_{work} / R_{free}	0.157 / 0.195
No. atoms	2682
Protein	2317
Ligands	126
Solvent	297
Protein residues	298
B-factors	18.33
Protein	16.80
Ligands	29.83
Water	27.68
RMS deviations	
Bond length (Å)	0.011
Bond angles (°)	1.24
MolProbity score	1.39

*Statistics for the highest-resolution shell are shown in parentheses.

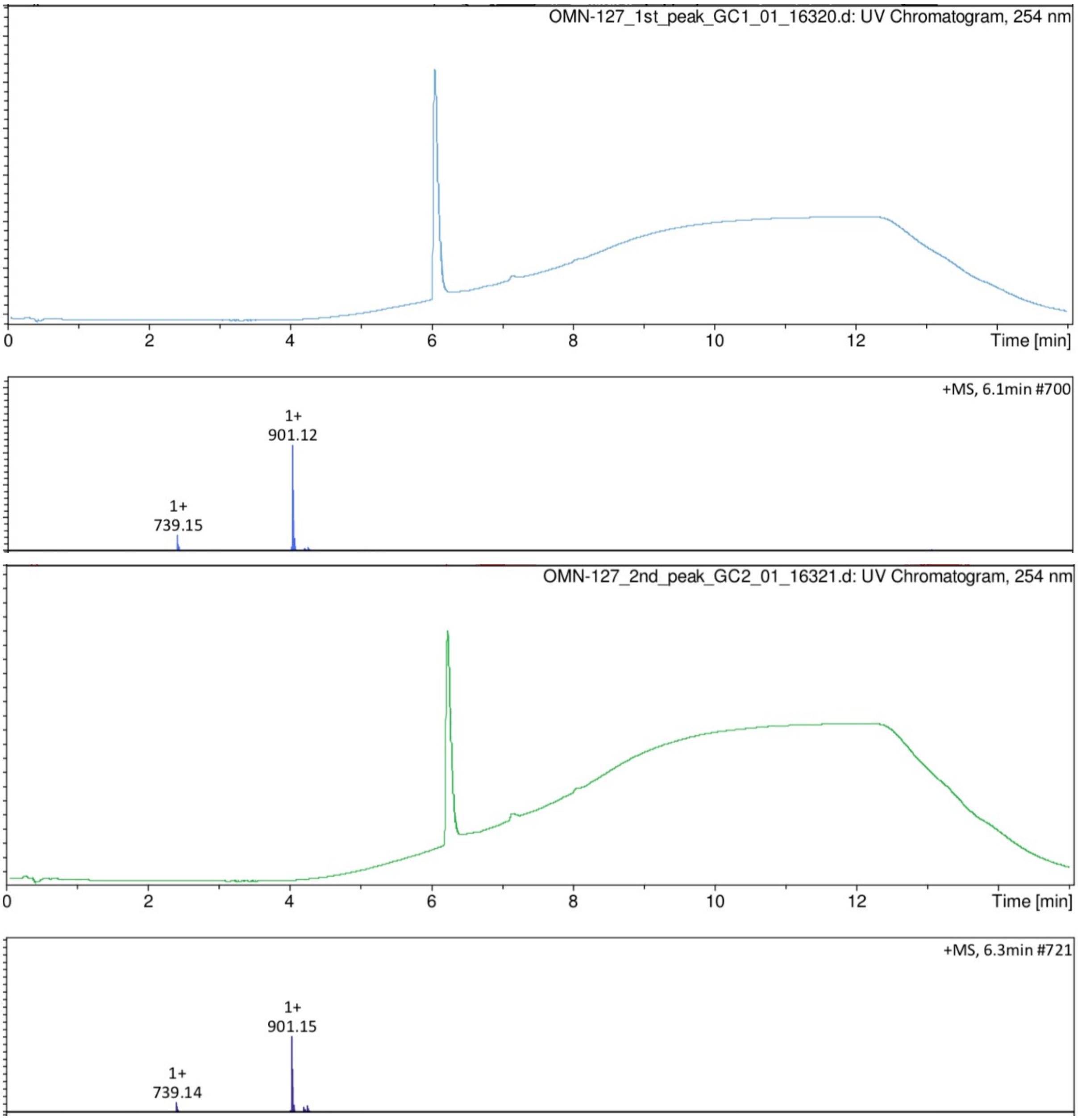

Figure S1. HPLC analysis of compounds **26a** (top) and **26b** (bottom).

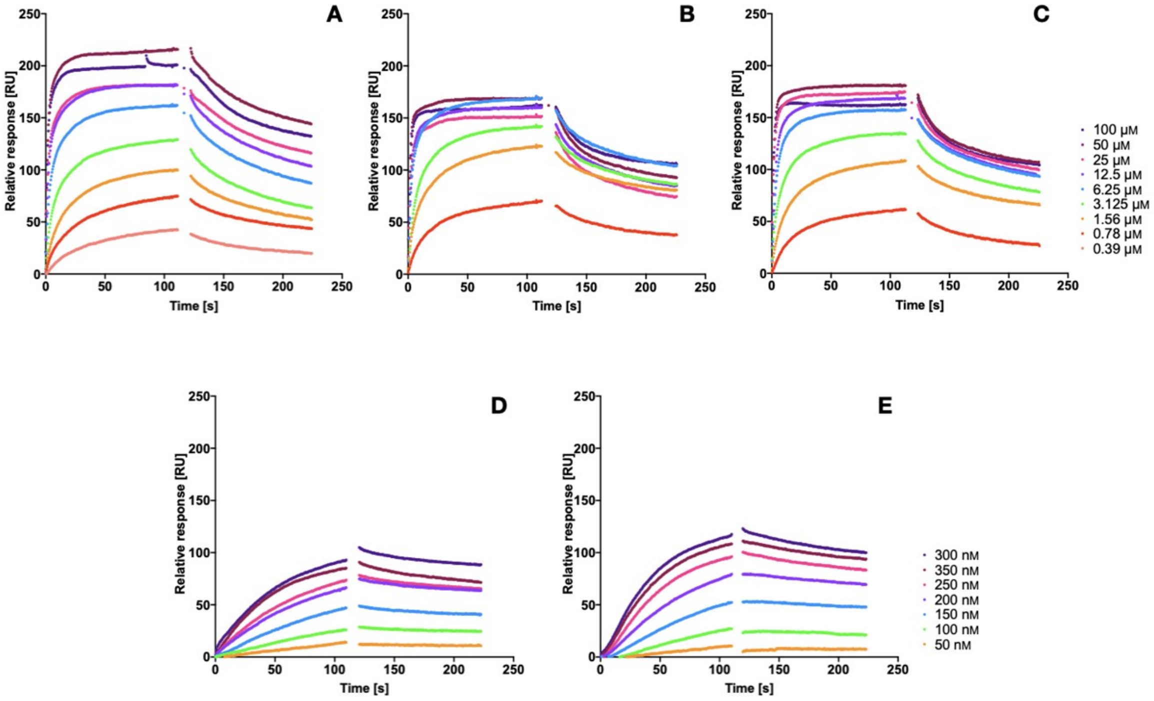

Figure S2. SPR binding curves of the interaction of dual inhibitors A) **24**, B) **18**, C) **25**, D) **27**, E) **26b** with LecA.

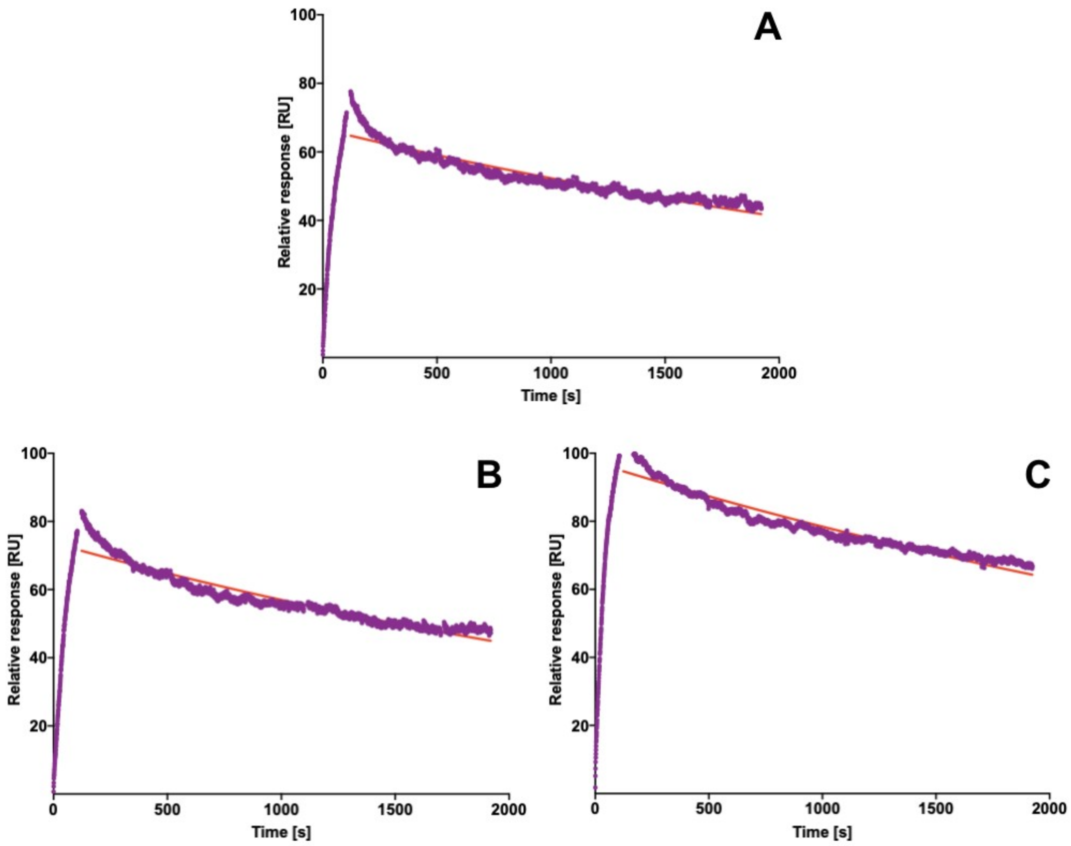

Figure S3. SPR dissociation kinetic analysis of the interaction of divalent LecA inhibitors A) **27**, B) **26a**, and C) **26b** with LecA.

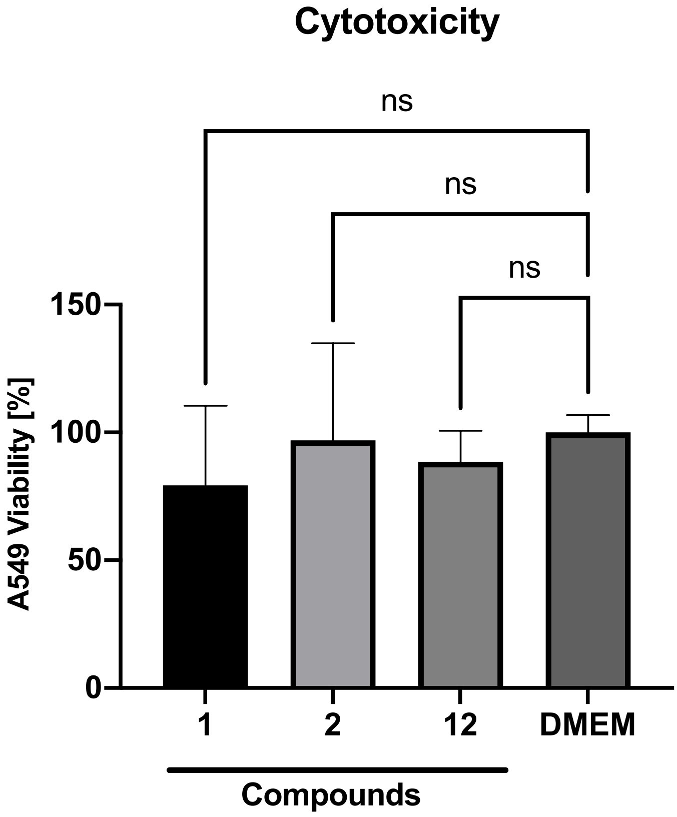

Figure S4. Evaluation of the cytotoxicity of compounds **1**, **2** and **12** at 100 µM. The graphs represent three independent experiments ± SD. One-way ANOVA statistical analysis was performed following Dunnett's multiple comparisons test, comparing the mean value of each concentration to the mean value of DMEM.

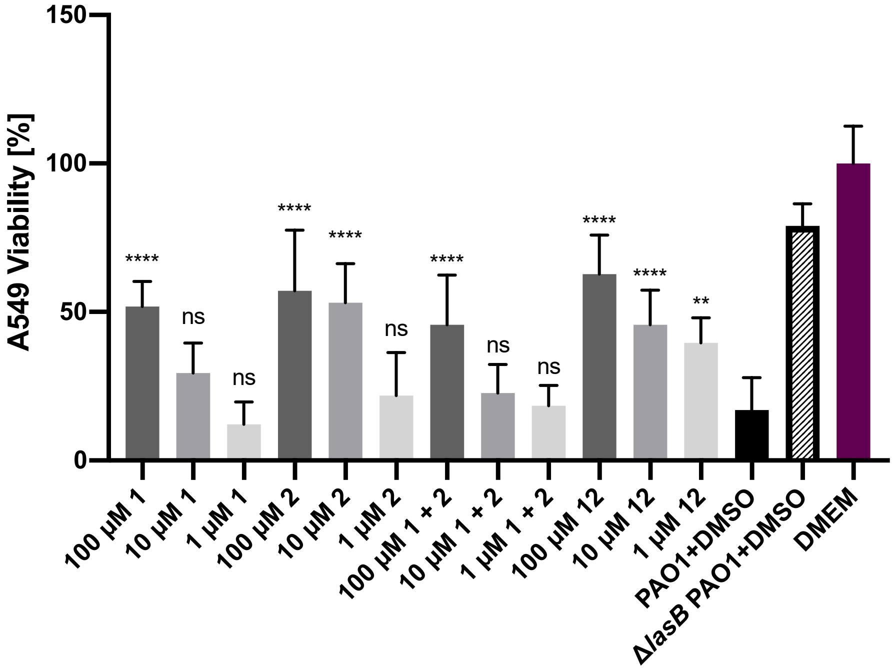

Figure S5. Inhibition of LasB-dependent cytotoxicity: The dose-response inhibitory effect of compounds **1**, **2** the combination of **1** and **2** and compound **12** against 10% (v/v) *P. aeruginosa* PAO1 culture supernatant. The graphs represent three independent experiments ± SD. One-way ANOVA statistical analysis was performed following Dunnett's multiple comparisons test, comparing the mean value of each concentration to the mean value of PAO1 without any treatment with compounds (**** $p \leq 0.0001$, ** $p \leq 0.01$).

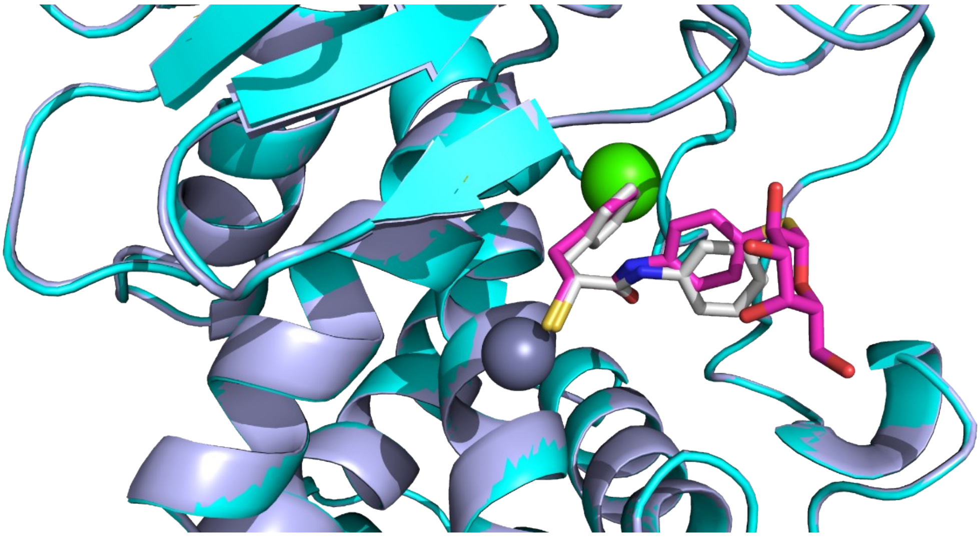

Figure S6. Superposition of the LasB-compound **11** (magenta) and LasB-α-alkyl-*N*-aryl mercaptoacetamide (gray; PDB 7OC7); Zn^{2+} is shown as a gray sphere; Ca^{2+} is shown as a green sphere; the protein structure demonstrates no major deviations between the two complexes

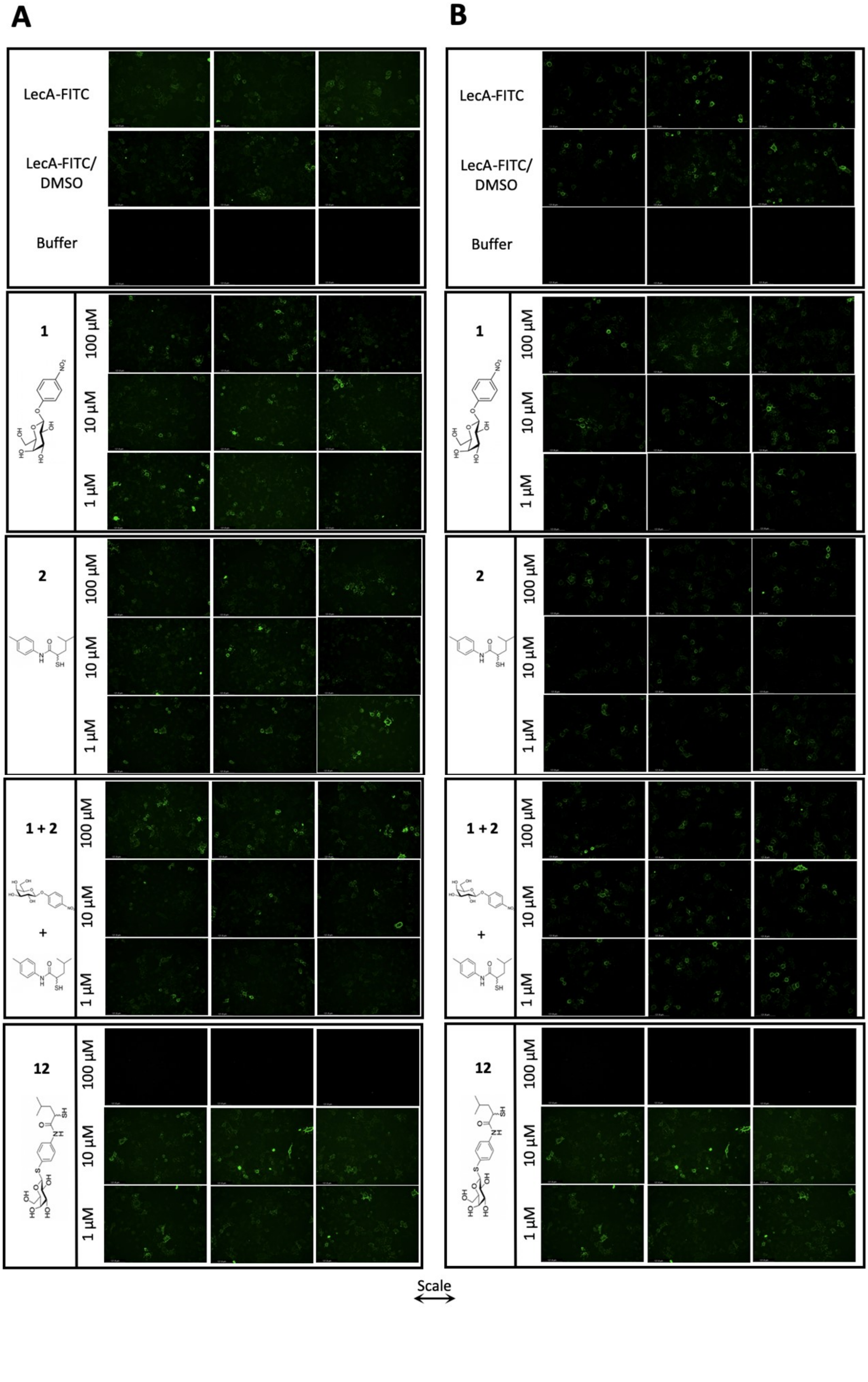

Figure S7. Fluorescence images of LecA-FITC bound to A549 cells in the presence of different concentrations of **1**, **2**, **1+2** and **12** (three representative images from two biological replicates (A, B). Scale bar corresponds to 250 µm

Supporting Information References

1 I. Joachim, S. Rikker, D. Hauck, D. Ponader, S. Boden, R. Sommer, L. Hartmann and A. Titz, *Org. Biomol. Chem.*, 2016, **14**, 7933–7948.
2 A. M. Kany, A. Sikandar, J. Haupenthal, S. Yahiaoui, C. K. Maurer, E. Proschak, J. Köhnke and R. W. Hartmann, *ACS Infect. Dis.*, 2018, **4**, 988–997.
3 R. Sommer, S. Wagner, A. Varrot, C. M. Nycholat, A. Khaledi, S. Häussler, J. C. Paulson, A. Imberty and A. Titz, *Chem. Sci.*, 2016, **7**, 4990–5001.
4 G. Beshr, A. Sikandar, E.-M. Jemiller, N. Klymiuk, D. Hauck, S. Wagner, E. Wolf, J. Koehnke and A. Titz, *Journal of Biological Chemistry*, 2017, **292**, 19935–19951.
5 E. Schönauer, A. M. Kany, J. Haupenthal, K. Hüsecken, I. J. Hoppe, K. Voos, S. Yahiaoui, B. Elsässer, C. Ducho, H. Brandstetter and R. W. Hartmann, *J. Am. Chem. Soc.*, 2017, **139**, 12696–12703.
6 J. Haupenthal, C. Baehr, S. Zeuzem and A. Piiper, *International Journal of Cancer*, 2007, **121**, 206–210.
7 W. A. M. Elgaher, M. Fruth, M. Groh, J. Haupenthal and R. W. Hartmann, *RSC Adv.*, 2014, **4**, 2177–2194.
8 M. D. Winn, C. C. Ballard, K. D. Cowtan, E. J. Dodson, P. Emsley, P. R. Evans, R. M. Keegan, E. B. Krissinel, A. G. W. Leslie, A. McCoy, S. J. McNicholas, G. N. Murshudov, N. S. Pannu, E. A. Potterton, H. R. Powell, R. J. Read, A. Vagin and K. S. Wilson, *Acta Crystallogr D Biol Crystallogr*, 2011, **67**, 235–242.
9 D. Liebschner, P. V. Afonine, M. L. Baker, G. Bunkóczi, V. B. Chen, T. I. Croll, B. Hintze, L. W. Hung, S. Jain, A. J. McCoy, N. W. Moriarty, R. D. Oeffner, B. K. Poon, M. G. Prisant, R. J. Read, J. S. Richardson, D. C. Richardson, M. D. Sammito, O. V. Sobolev, D. H. Stockwell, T. C. Terwilliger, A. G. Urzhumtsev, L. L. Videau, C. J. Williams and P. D. Adams, *Acta Crystallogr D Struct Biol*, 2019, **75**, 861–877.
10 P. Emsley and K. Cowtan, *Acta Crystallogr D Biol Crystallogr*, 2004, **60**, 2126–2132.
11 L. Schrödinger and W. DeLano, PyMOL 2020.
12 R. B. Cohen, S. H. Rutenburg, K.-C. Tsou, M. A. Woodbury and A. M. Seligman, *Journal of Biological Chemistry*, 1952, **195**, 607–614.
13 S. Escopy, Y. Singh and A. V. Demchenko, *Org. Biomol. Chem.*, 2019, **17**, 8379–8383.
14 F. Casoni, L. Dupin, G. Vergoten, A. Meyer, C. Ligeour, T. Géhin, O. Vidal, E. Souteyrand, J.-J. Vasseur, Y. Chevolot and F. Morvan, *Org Biomol Chem*, 2014, **12**, 9166–9179.
15 T. Fujisawa, K. Igeta, S. Odake, Y. Morita, J. Yasuda and T. Morikawa, *Bioorganic & Medicinal Chemistry*, 2002, **10**, 2569–2581.
